# Negative electrostatic potentials in a Hofmann-type metal-organic framework for efficient acetylene separation

**DOI:** 10.1038/s41467-022-33271-3

**Published:** 2022-09-20

**Authors:** Yuan Liu, Junhui Liu, Hanting Xiong, Jingwen Chen, Shixia Chen, Zheling Zeng, Shuguang Deng, Jun Wang

**Affiliations:** 1 School of Chemistry and Chemical Engineering, Nanchang University, Nanchang, Jiangxi 330031 China; 2 School for Engineering of Matter, Transport and Energy, Arizona State University, Tempe, Arizona 85287 USA

**Keywords:** Chemistry, Materials science

## Abstract

Efficient adsorptive separation of acetylene (C_2_H_2_) from carbon dioxide (CO_2_) or ethylene (C_2_H_4_) is industrially important but challenging due to the identical dynamic diameter or the trace amount. Here we show an electrostatic potential compatible strategy in a nitroprusside-based Hofmann-type metal-organic framework, Cu(bpy)NP (NP = nitroprusside, bpy = 4,4’-bipyridine), for efficient C_2_H_2_ separation. The intruding cyanide and nitrosyl groups in undulating one-dimensional channels induce negative electrostatic potentials for preferential C_2_H_2_ recognition instead of open metal sites in traditional Hofmann-type MOFs. As a result, Cu(bpy)NP exhibits a 50/50 C_2_H_2_/CO_2_ selectivity of 47.2, outperforming most rigid MOFs. The dynamic breakthrough experiment demonstrates a 99.9% purity C_2_H_4_ productivity of 20.57 mmol g^−1^ from C_2_H_2_/C_2_H_4_ (1/99, *v*/*v*) gas-mixture. Meanwhile, C_2_H_2_ can also be captured and recognized from ternary C_2_H_2_/CO_2_/C_2_H_4_ (25/25/50, *v/v/v*) gas-mixture. Furthermore, computational studies and in-situ infrared spectroscopy reveal that the selective C_2_H_2_ binding arises from the compatible pore electro-environment generated by the electron-rich N and O atoms from nitroprusside anions.

## Abstract (web summary)

Separating acetylene from carbon dioxide or ethylene is challenging due to near identical molecular diameters or the trace amounts. Here authors prepare nitroprusside-based Hofmann-type MOFs for efficient C_2_H_2_ separation and recognition.

## Introduction

Acetylene (C_2_H_2_) capture from gas-mixtures is of great industrial importance^1,2^. As a vital chemical feedstock and industrial gas^3^, C_2_H_2_ is typically generated by the partial combustion of methane, a considerable amount of carbon dioxide (CO_2_) inevitably coexists in the crude products. C_2_H_2_ and CO_2_ molecules both possess linear configurations with subtle differences in molecular dimensions (3.3 × 3.3 × 5.7 Å for C_2_H_2_ and 3.2 × 3.3 × 5.4 Å for CO_2_), and show close similarities in physical properties, including quadrupole moments, boiling point, and polarity (Supplementary Table 1)^4,5^. On the other hand, the highly reactive C_2_H_2_ can induce undesirable side effects in various industrial operations. For instance, in ethylene (C_2_H_4_) manufacturing by the steam cracking of naphtha or dehydrogenation of ethane^6,7^, the trace amount of C_2_H_2_ (1%) must be removed to an acceptable level of 40 ppm, otherwise the residue C_2_H_2_ will deactivate the Ziegler–Natta catalyst and lower the quality of polyethylene commodities^8^. In industry, trace C_2_H_2_ removal is achieved by partial hydrogenation or solvent absorption, which are energy- and cost-intensive^9^. Therefore, alternative technologies with high capital efficiency have attracted great interest in separating C_2_H_2_ from CO_2_ and C_2_H_4_ due to their industrial relevance and scientific challenge, such as adsorptive separations^10^.

**Fig. 1:**
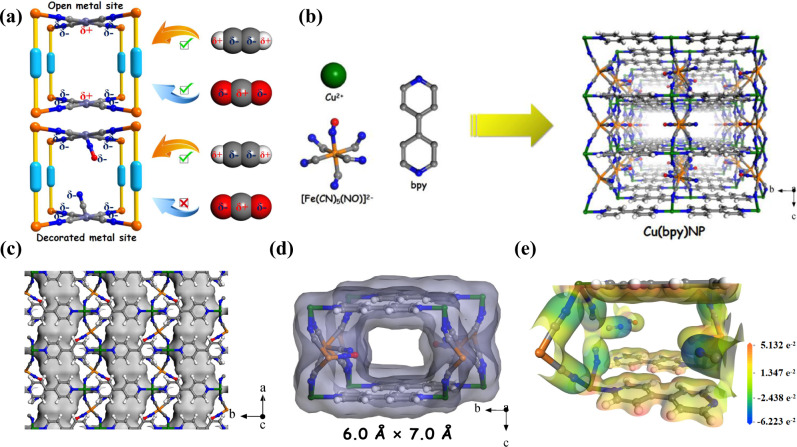
Material synthesis and crystallographic structures. Schematic illustration of **a** traditional and nitroprusside-based Hofmann-type MOF networks and **b** the building blocks (Cu^2+^, [Fe(CN)_5_(NO)]^2−^, and bpy) and the 3D ***sqc***-net topology of Cu(bpy)NP; **c** view of the pore channels by the Connolly surface along the *ab*-plane, **d** illustration of the channel size along the *bc*-plane, and **e** the ESP mapping for the channel of Cu(bpy)NP (color code: C, gray; H, white; N, blue; O, red; Cu, green; Fe, orange).

Since the discovery of Prussian blue in 1704, transition metal cyanide complexes, also referred to as cyanometallates, have been extensively synthesized^11^. The strong basicity of cyanide, as *δ*-donor and *π*-acceptor, endows the ability and versatility of coordination with most transition metals^12^. Particularly, cyanide-based metal-organic frameworks (MOFs) with interior vacancies and open spaces emerge as an intriguing class of solid adsorbents^13,14^. As a family of Prussian blue analogs, Hofmann-type MOFs have a general formula of M(pyz)[M’(CN)]_4_, where M and M’ are divalent metal ions and pyz is the pyrazine bidentate organic ligand^15^. Besides the confined pore spaces, another prevailing feature is the existence of abundant open metal sites (OMSs) incorporated in cyanometallate ligand that can significantly enhance the C_2_H_2_ capacity through strong electrostatic interactions. For example, Co(pyz)[Ni(CN)]_4_, termed as ZJU-74a, exhibited the record C_2_H_2_ adsorption capacity of 49 cm^3^ g^−1^ at 0.01 bar and 296 K due to the sandwich-like open Ni sites from adjacent [Ni(CN)]_4_^2−^ ligands^13^. However, the open Ni sites can also interact with the electronegative O atoms of CO_2_ (Fig. 1a), as evidenced by the close C_2_H_2_ and CO_2_ capacity (3.82 mmol g^−1^ vs 3.13 mmol g^−1^) at 1.0 bar and 296 K.

Nitroprusside is a unique type of cyanide ligand in which the Fe(II) atoms are octahedrally coordinated by five carbon atoms from independent cyanide groups and one nitrogen atom from a nitrosyl group (Fig. 1b). The straightforward coordination of Cd^2+^ and Ni^2+^ ions with nitroprusside (NP) have yielded Cd-NP and Ni-NP that showed superior CO_2_/C_2_H_2_ and C_3_H_6_/C_3_H_8_ separation performances^11,16^, respectively. Nevertheless, to the best of our knowledge, the successful preparation of Hofmann-type MOFs using nitroprusside ligand has never been reported. Unlike the directly exposed OMSs in cyanometallate ligand, the intruding cyanide and nitrosyl groups can create a negative-charged electrostatic potential field for preferred C_2_H_2_ capture, because C_2_H_2_ possesses a positive charge distribution at both ends (Fig. 1a)^14^. Note that creating a negative-charged pore environment is challenging due to the limited choice of suitable functional groups.

Herein, we report a Hofmann-type MOFs employing nitroprusside ligand, Cu(bpy)NP (NP = nitroprusside, bpy = 4,4’-bipyridine), for efficient and selective capture of C_2_H_2_ from C_2_H_2_/CO_2_ and C_2_H_2_/C_2_H_4_ gas-mixtures. In particular, Cu(bpy)NP exhibits a three-dimensional ***sqc*** topology with a suitable cavity size of 6.0 × 7.0 Å^2^. The incorporation of cyanide and nitrosyl groups on the pore walls afford a complementary negative electrostatic potential field for C_2_H_2_ binding. As a result, Cu(bpy)NP shows a large C_2_H_2_ capacity (50.7 cm^3^ g^−1^) and superior C_2_H_2_/CO_2_ (47.2) and C_2_H_2_/C_2_H_4_ (28.5) selectivity at 298 K and 1.0 bar. Dynamic breakthrough experiments confirm its practical C_2_H_2_ capture performances from binary C_2_H_2_/CO_2_ and C_2_H_2_/C_2_H_4_ gas-mixtures and ternary C_2_H_2_/CO_2_/C_2_H_4_ gas-mixture. Cu(bpy)NP affords C_2_H_4_ productivity of 20.57 mmol g^−1^ from C_2_H_2_/C_2_H_4_ (1/99, *v/v*) gas-mixture. Theoretical modeling and in-situ IR studies reveal that C_2_H_2_ molecules are collectively bound by two nitrosyl groups and one cyanide group via multiple strong interactions.

## Results

### Synthesis and characterization

By dripping the aqueous solution of Cu(NO_3_)_2_·3H_2_O and sodium nitroprusside (NP) into a methanol solution of 4,4’-bipyridine (bpy), the Cu(bpy)NP can be obtained under mild conditions with a yield of 67% (Fig. 1b). Cyan square-block single-crystals of Cu(bpy)NP were also successfully prepared by the slow-diffusion method (Supplementary Fig. 1). Single-crystal X-ray diffraction studies revealed that Cu(bpy)NP crystallizes in an *orthorhombic* space group *Pmma* and adopts a 4,6-connected ***sqc*** topology (Fig. 1b, CCDC: 2124121). Each Cu moiety is octahedrally coordinated by two ditopic bridging bpy linkers at the axial positions and four independent cyanide groups from [Fe(CN)_5_(NO)]^2−^ anions at equatorial positions (Supplementary Fig. 2). In the structure of Cu(bpy)NP, the undulating two-dimensional (2D) channels across the *ab*-plane are different from the regular one-dimensional (1D) channels in previously reported Hofmann-type MOFs (Fig. 1c)^12,13^. The channels in Cu(bpy)NP showed a channel dimension of 6.0 × 7.0 Å^2^ along the *bc*-plane (Fig. 1d). The volume of its solvent-accessible void was calculated to be 34.4% of the total volume. Moreover, the electrostatic potential (ESP) of channel interior in Cu(bpy)NP was mapped by density functional theory (DFT) calculations (Fig. 1e), and strong negative potentials were disclosed near the nitrogen atom in the cyanide group and oxygen atom in the nitrosyl group that are compatible for C_2_H_2_ adsorption, however, which are mutually repulsive to CO_2_ with negatively charged ends (Supplementary Fig. 3).

The phase purity of the as-synthesized Cu(bpy)NP was verified by the powder X-ray diffraction (PXRD) pattern, which is consistent with the simulated one, while no noticeable change was observed on the activated sample (Supplementary Fig. 4 Supplementary Table 2). Furthermore, the Rietveld refinement of PXRD revealed that the activated Cu(bpy)NP crystallized in the same crystal system and showed similar cell parameters with the as-synthesized sample, indicating its rigid framework after solvent removal (Supplementary Fig. 5 and Supplementary Table 3). The PXRD patterns of C_2_H_2_-loaded samples under different pressures (0–1.0 bar) at 298 K showed inconspicuous changes in position and intensity of diffraction peaks, manifesting structural robustness during adsorption processes (Supplementary Fig. 6). The structural integrity was examined by immersing Cu(bpy)NP in various organic solvents for 1 week and boiling water for 3 h, intact PXRD patterns can be maintained, suggesting its good chemical stability (Supplementary Fig. 7a). Meanwhile, Cu(bpy)NP could survive wide range acid/basic solutions, whereas the crystal structure was destroyed at extreme conditions, i.e., pH = 13 and 1 (Supplementary Fig. 7b). TGA revealed a weight loss of 2.9% at 220 °C, corresponding to the removal of guest molecules (Supplementary Fig. 8). The Brunauer-Emmett-Teller (BET) surface area of Cu(bpy)NP was measured to be 459 m^2^ g^−1^ with a total pore volume of 0.45 cm^3^ g^−1^ by CO_2_ adsorption at 195 K (Supplementary Fig. 9). The pore size was determined to center at 6.6 Å, consistent with the channel size derived from the crystal structure. The pore properties were also probed by N_2_ at 77 K (Supplementary Fig. 10), the decreased BET specific surface area of 121 m^2^ g^−1^ can be attributed to the larger size and property inertness of N_2_ as the probe molecule.

### Adsorption and separation performances

**Fig. 2:**
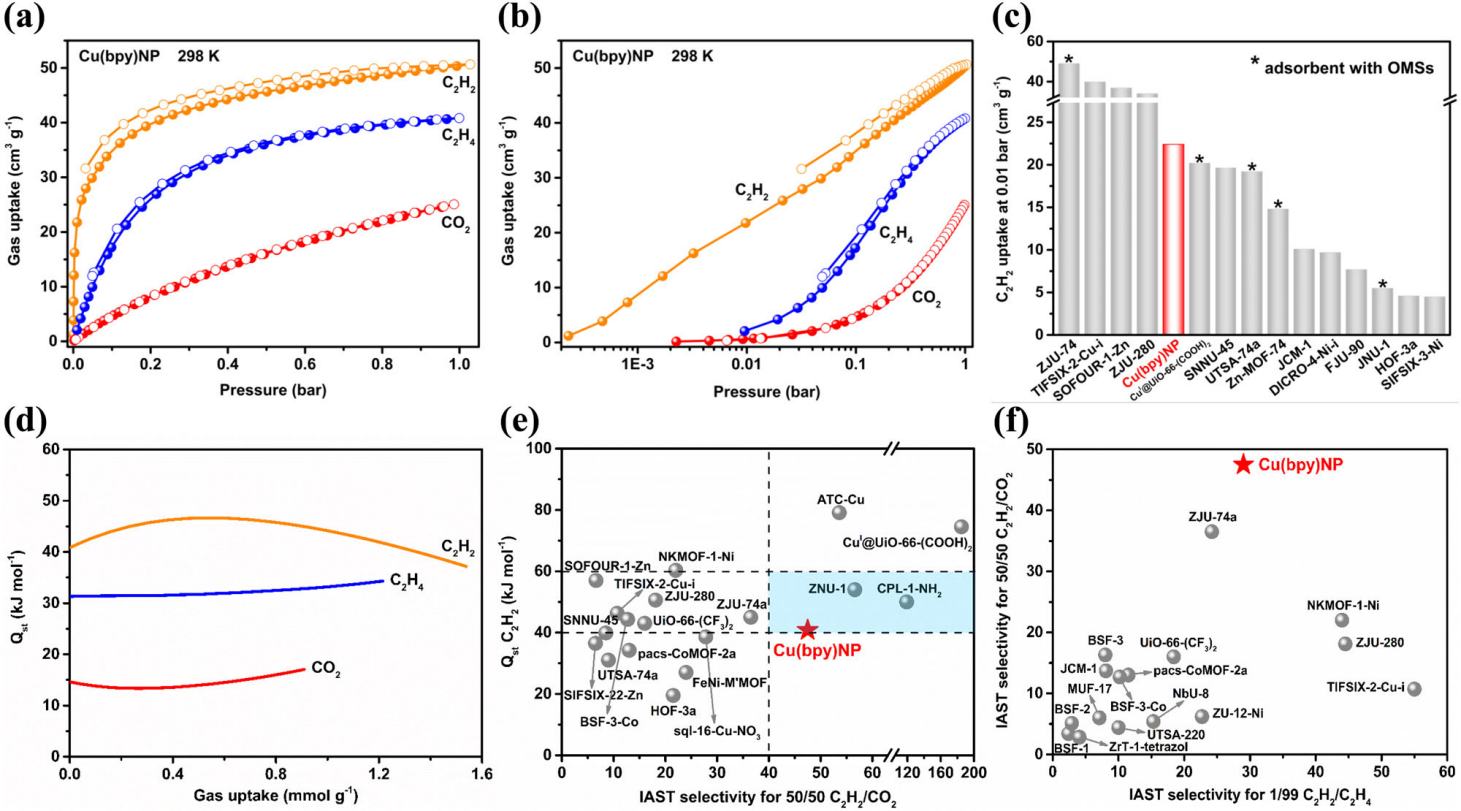
C_2_H_2_, CO_2_, and C_2_H_4_ sorption in nitroprusside-based Hofmann-type MOFs. C_2_H_2_, CO_2_, and C_2_H_4_ adsorption isotherms **a** in linear form and **b** in logarithmic form of Cu(bpy)NP at 298 K; **c** comparison of C_2_H_2_ uptakes with best-performing materials at 0.01 bar and 298 K; **d** *Q*_*st*_ plots of C_2_H_2_, CO_2_, and C_2_H_4_ on Cu(bpy)NP; **e** comparison of C_2_H_2_ *Q*_*st*_ and 50/50 C_2_H_2_/CO_2_ IAST selectivity with leading adsorbents; **f** comparison of 50/50 C_2_H_2_/CO_2_ and 1/99 C_2_H_2_/C_2_H_4_ IAST selectivity of Cu(bpy)NP with reported adsorbents at 298 K and 1.0 bar.

Single-component gas adsorption isotherms of C_2_H_2_, CO_2_, and C_2_H_4_ were collected at 273, 298, and 323 K, respectively (Fig. 2a, Supplementary Fig. 11, and Supplementary Tables 6-8). Cu(bpy)NP exhibited a higher C_2_H_2_ adsorption capacity (50.7 cm^3^ g^−1^) than that of C_2_H_4_ (40.8 cm^3^ g^−1^) and CO_2_ (25.1 cm^3^ g^−1^) at 298 K and 1.0 bar. Approximately, two C_2_H_2_, one CO_2_, and one and a half C_2_H_4_ molecules are adsorbed per unit cell, respectively. Generally, the gas separation performances greatly correlate with the gas adsorption behaviors in low-pressure ranges, in which the adsorbent-adsorbate affinity dominates^17^. As shown in Fig. 2b, Cu(bpy)NP displayed a C_2_H_2_ uptake of 22.4 cm^3^ g^−1^ at 0.01 bar and 298 K, notably, even comparable to adsorbents with OMSs, including Cu^I^@UiO-66-(COOH)_2_ (20.2 cm^3^ g^−1^)^18^, UTSA-74a (19.2 cm^3^ g^−1^)^19^, Zn-MOF-74 (14.8 cm^3^ g^−1^)^20^, and JNU-1 (5.5 cm^3^ g^−1^)^21^ (Fig. 2c and Supplementary Table 9). In sharp contrast, the adsorption uptake of C_2_H_4_ (2.1 cm^3^ g^−1^) and CO_2_ (0.6 cm^3^ g^−1^) was almost negligible at 0.01 bar. The adsorption isotherms have experimentally confirmed the concept that the negative electrostatic potential can selectively adsorb C_2_H_2_ over CO_2_ and C_2_H_4_.

To evaluate the affinity of Cu(bpy)NP toward C_2_H_2_, CO_2_, and C_2_H_4_, the isosteric enthalpy of adsorption (*Q*_st_) was calculated by Virial fitting from adsorption isotherms at three temperatures (Supplementary Fig. 12 and Supplementary Table 10). The calculated *Q*_st_ for C_2_H_2_ is 40.8 kJ mol^−1^, higher than that of CO_2_ (14.6 kJ mol^−1^) and C_2_H_4_ (31.3 kJ mol^−1^) at near zero coverage (Fig. 2d). The moderate *Q*_st_ for C_2_H_2_ indicates both strong binding and energy-efficient regeneration (Fig. 2e)^22^. Moreover, the difference in *Q*_*st*_ (Δ*Q*_*st*_) represents the binding selectivity of adsorbents, the Δ*Q*_*st*_ for C_2_H_2_ and CO_2_ is calculated to be 26.2 kJ mol^−1^, which is the most prominent except for two MOFs with rich OMSs (Supplementary Fig. 17), i.e., Cu^I^@UiO-66-(COOH)_2_ (45.6)^18^ and ATC-Cu (42.6)^23^.

The ideal adsorbed solution theory (IAST) was employed to evaluate the adsorptive separation selectivity, and the adsorption isotherm fittings by the dual-site Langmuir-Freundlich model were shown in Supplementary Figs. 13–15 and Supplementary Table 11. First, the adsorption kinetics of C_2_H_2_, CO_2_, and C_2_H_4_ were evaluated on Cu(bpy)NP, which depicted that the equilibrium times for all gases were about 6 min at 298 K and 0.4 bar (Supplementary Fig. 16). Benefiting from the preferred C_2_H_2_ adsorptions at low pressures, Cu(bpy)NP exhibited a benchmark 50/50 C_2_H_2_/CO_2_ selectivity of 343.8 at 0.01 bar and 298 K, and gradually decreased to 47.2 at 1.0 bar (Supplementary Fig. 18). Notably, such C_2_H_2_/CO_2_ selectivity at ambient conditions, to our knowledge, is only lower than benchmark adsorbents such as Cu^I^@UiO-66-(COOH)_2_ (185)^18^, CPL-1-NH_2_ (119)^24^, ZNU-1 (56.6)^25^, and ATC-Cu (53.6)^24^, and higher than leading materials including ZJU-74a (36.5)^13^, NKMOF-1-Ni (22.0)^17^, and HOF-3a (21.5)^26^ (Fig. 2e). It is worth pointing out that Cu(bpy)NP should be considered as one of the most promising candidates that show favorable C_2_H_2_/CO_2_ selectivity (>40) and suitable C_2_H_2_ *Q*_st_ for regeneration (40~60 kJ mol^−1^)^22^. Similarly, the 1/99 C_2_H_2_/C_2_H_4_ IAST selectivity of 781.2 and 28.5 was obtained at 0.01 bar and 1.0 bar and 298 K, respectively (Supplementary Fig. 19a). Besides the advantageous C_2_H_2_/C_2_H_4_ IAST selectivity (Supplementary Fig. 19b and Supplementary Table 9), noticeably, the C_2_H_2_/CO_2_ IAST selectivity on Cu(bpy)NP was also superior to many reported adsorbents (Fig. 2f).

### Transient breakthrough experiments

**Fig. 3:**
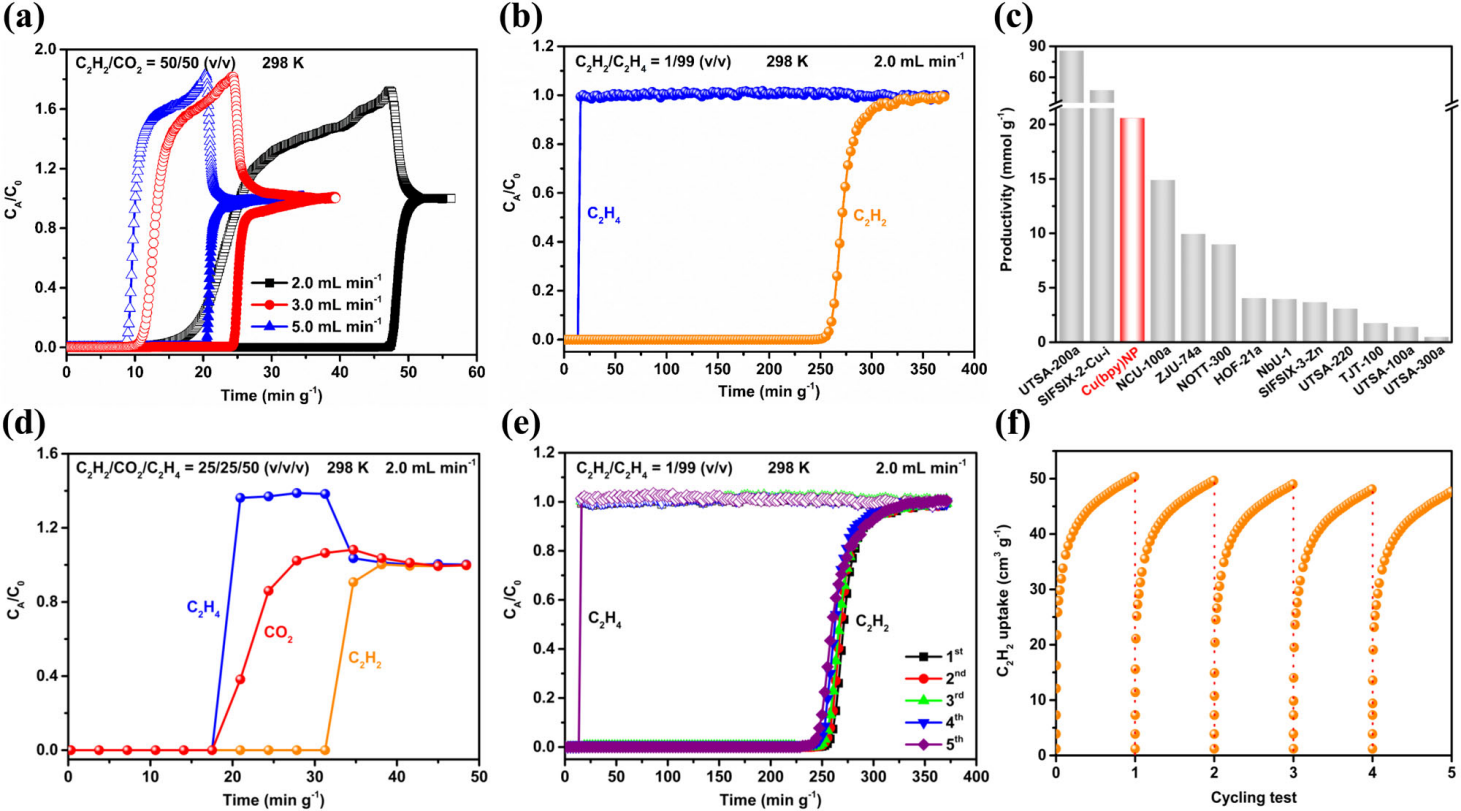
C_2_H_2_, CO_2_, and C_2_H_4_ separation performances. Dynamic breakthrough curves of Cu(bpy)NP for **a** C_2_H_2_/CO_2_ (50/50, *v/v*) and **b** C_2_H_2_/C_2_H_4_ (1/99, *v/v*) gas-mixtures; **c** comparison of C_2_H_4_ productivity with representative MOFs; **d** dynamic breakthrough curve of Cu(bpy)NP for ternary C_2_H_2_/CO_2_/C_2_H_4_ (25/25/50, *v/v/v*) gas-mixture; **e** cycling dynamic breakthrough tests for C_2_H_2_/C_2_H_4_ (1/99, *v/v*) on Cu(bpy)NP; **f** continuous five C_2_H_2_ adsorption isotherms of Cu(bpy)NP at 298 K.

To further experimentally validate the separation performances of Cu(bpy)NP, dynamic breakthrough experiments were carried out under ambient conditions. As shown in Fig. 3a, Cu(bpy)NP can efficiently separate an equimolar gas-mixture of C_2_H_2_/CO_2_, at a flow rate of 2.0 mL min^−1^, CO_2_ was detected first at the outlet of the separation bed at around 20 min, while C_2_H_2_ was not eluted from the column until 78 min. The separation selectivity (*α*_AC_) for equimolar C_2_H_2_/CO_2_ mixture was calculated to be 3.9 on Cu(bpy)NP based on the breakthrough curve, outperforming SNNU-45 (2.9)^27^, NKMOF-1-Ni (2.6)^17^, HOF-3a (2.0)^27^, and FeNi-M’MOF (1.7)^14^ (Supplementary Fig. 20). When the flow rate increased to 3.0 and 5.0 mL min^−1^, the breakthrough time decreased as expected but good separation effects remained. Meanwhile, as illustrated by the desorption curves, CO_2_ desorbed quickly in 10 min and the complete desorption of C_2_H_2_ was achieved at 90 min, indicating a long operation window for C_2_H_2_ collection with high C_2_H_2_ purity above 99% (Supplementary Fig. 21). As for C_2_H_2_/C_2_H_4_ (1/99, *v/v*) gas-mixture, C_2_H_4_ immediately broke through the packed column with Cu(bpy)NP at 22 min, while C_2_H_2_ was trapped and eluted until 400 min (Fig. 3b). The C_2_H_4_ productivity with 99.9 + % purity was calculated to be 20.57 mmol g^−1^ on Cu(bpy)NP, which is only lower than that of SIFSIX-2-Cu-i (47.4 mmol g^−1^)^9^ and UTSA-200a (85.7 mmol g^−1^)^28^ (Fig. 3c). Even when the concentration of C_2_H_2_ increased to 50%, Cu(bpy)NP also maintained the efficient and clean separation for C_2_H_2_/C_2_H_4_ (50/50, *v/v*) gas-mixture (Supplementary Fig. 22).

Furthermore, we also carried out the dynamic breakthrough experiment with ternary C_2_H_2_/CO_2_/C_2_H_4_ (25/25/50, *v/v/v*) gas-mixture. As shown in Fig. 3d, CO_2_ and C_2_H_4_ concurrently broke through the adsorption column at 28 min, whereas C_2_H_2_ was not detected at the exit until 50 min. The cycling and regeneration capabilities of Cu(bpy)NP are important parameters for practical industrial applications. Therefore, cycling breakthrough cycle experiments for both C_2_H_2_/CO_2_ (50/50, *v/v*) and C_2_H_2_/C_2_H_4_ (1/99, *v/v*) were conducted (Fig. 3e and Supplementary Fig. 23), during five continuous cycles, no noticeable decay in the residual time was observed. Also, five successive C_2_H_2_ adsorption-desorption isotherms showed intact C_2_H_2_ uptakes, manifesting the excellent reusability of Cu(bpy)NP (Fig. 3f).

### In-situ IR and modeling simulation studies

**Fig. 4:**
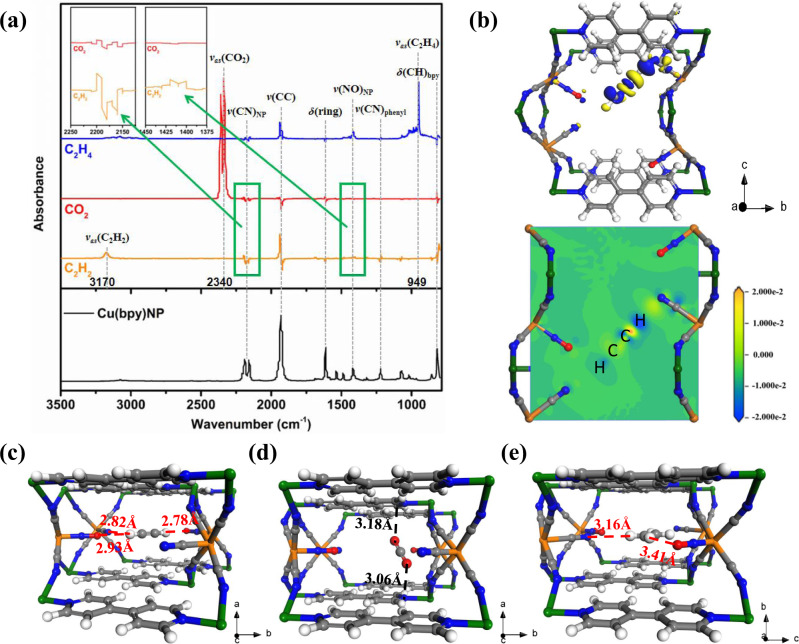
In-situ IR spectra and binding sites for C_2_H_2_, CO_2_, and C_2_H_4_ in Cu(bpy)NP. **a** In-situ IR spectra of gas-loaded Cu(bpy)NP samples; **b** Charge density difference plots showing the interaction between C_2_H_2_; DFT-D calculated binding sites of **c** C_2_H_2_, **d** CO_2_, and **e** C_2_H_4_ in Cu(bpy)NP. The distances include the Van der Waals radius of atoms (Color code: C, gray; H, white; N, blue; O, red; Cu, green; Fe, orange).

In-situ infrared (IR) spectroscopic measurements were conducted on gas-loaded samples to illustrate the interactions between three adsorbates and Cu(bpy)NP. It is recognized that acidic C_2_H_2_ molecules tend to form strong interactions with basic sites^8,29^. As shown in Fig. 4a, the *ν*_as_(C_2_H_2_) stretching band of adsorbed C_2_H_2_ down-shifted to 3170 cm^−1^ with reference to the gas-phase value at 3287 cm^−1^, indicating the existence of guest–host interactions with possible charge transfers^30–32^. The strong perturbation of characteristic bands, i.e., ν(CN)_NP_, *ν*(CC)_bpy_, *ν*(CN)_bpy_, *δ*(CH)_bpy_, *ν*(NO)_NP_, and *δ*(ring), suggested that C_2_H_2_ also interacted with Cu(bpy)NP framework via multiple weak interactions. The adsorbed CO_2_ within Cu(bpy)NP was confirmed by its asymmetric stretching band *ν*_as_(CO_2_) at 2340 cm^−1 ^^33^. Compared to the C_2_H_2_-loaded framework, the perturbations of characteristic bands were obviously decreased in intensity, especially for *ν*(CN)_NP_ and *ν*(NO)_NP_ (Fig. 4a inset), verifying that the complementary electrostatic potential created by cyanide and nitrosyl groups were responsible for selective C_2_H_2_ capture. As for C_2_H_4_, the *ν*_as_(C_2_H_4_) stretching band appeared at 949 cm^−1^ ^34,35^, and the interaction mode was similar to that of C_2_H_2_-loaded samples. It was noted that the *ν*(NO)_NP_ bond was intensified, indicating the favorable affinity toward nitrosyl groups.

To gain insight into the adsorption mechanisms of C_2_H_2_, CO_2_, and C_2_H_4_ in Cu(bpy)NP, theoretical molecular simulations using the grand canonical Monte Carlo (GCMC) and first-principles dispersion-corrected density functional theory (DFT-D) were carried out. The GCMC-simulated C_2_H_2_, CO_2_, and C_2_H_4_ adsorption isotherms agreed well with the experimental isotherms (Supplementary Fig. 24). The distribution densities were firstly investigated at 1 kPa, C_2_H_2_ molecules are mainly distributed in two locations near the cyanide and nitrosyl groups. In sharp contrast, the CO_2_ molecules are distributed in the middle of the channels that are apart from nitroprusside anions. Whereas, the C_2_H_4_ molecules occupy almost identical locations in the channels of Cu(bpy)NP but with less distribution density compared to C_2_H_2_ (Supplementary Fig. 25a–c). As the loading pressure increased to 100 kPa, the adsorption locations for each adsorbent remained, while the contribution density is in the order of C_2_H_2_ > C_2_H_4_ > CO_2_, which is consistent with the experimental adsorption capacities (Supplementary Fig. 25d–f).

We firstly carried out the charge transfer analysis on the gas-loaded structures by DFT calculations. As shown in Fig. 4b, the blue and yellow surfaces indicate charge accumulation and charge depletion, respectively. The close end-on adsorption configuration of H atom of C_2_H_2_ and N atom of cyanide groups implied the strong electro-field induced C_2_H_2_ capture (Fig. 4b). Moreover, the negatively charged H atom of C_2_H_2_, opposite to its original positive-charged state, revealed the charge transfer from Cu(bpy)NP framework to adsorbed C_2_H_2_. In contrast, CO_2_ and C_2_H_4_ molecules displayed the side-on adsorption configurations in Cu(bpy)NP skeleton, and no noticeable charge transfer was observed (Supplementary Fig. 26). These results highlighted the effect of negatively charged pore environment in enhancing the adsorption and recognition of C_2_H_2_.

The lowest-energy gas binding configurations of C_2_H_2_, CO_2_, and C_2_H_4_ in Cu(bpy)NP were also calculated. As shown in Fig. 4c, C_2_H_2_ was roughly oriented parallel to the bpy ligand along the *a*-axis, where it was captured by three independent [Fe(CN)_5_(NO)]^2−^ anions via strong interactions of two C-H•••O bonds (2.78–2.93 Å) and one C-H•••N bond (2.82 Å). The binding energy of C_2_H_2_ in Cu(bpy)NP was calculated to be 38.4 kJ mol^−1^, slightly lower than the experimental C_2_H_2_ *Q*_st_ (40.8 kJ mol^−1^). In contrast, CO_2_ was adsorbed by two opposite bpy ligands through weak interactions of N•••O(CO_2_) (3.06 Å) and C•••C(CO_2_) (3.18 Å) with a calculated CO_2_ binding energy of 13.4 kJ mol^−1^ (Fig. 4d). Clearly, the negative electrostatic potential generated by [Fe(CN)_5_(NO)]^2−^ anions induced the preferential binding with C_2_H_2_ over CO_2_. Similarly, Cu(bpy)NP displayed relatively weak interactions with C_2_H_4_ via the C-H•••N bond (3.16 Å) and C-H•••O bond (3.41 Å), the C_2_H_4_ binding energy was calculated to be 28.5 kJ mol^−1^ (Fig. 4e).

## Discussion

In summary, we synthesized and reported a nitroprusside-based Hofmann-type MOF adsorbent, Cu(bpy)NP, for simultaneous efficient separation of C_2_H_2_ from C_2_H_2_/CO_2_ and C_2_H_2_/C_2_H_4_ gas-mixtures. Compared to the traditional Hofmann-type MOFs with rich OMSs, the cyanide and nitrosyl groups of nitroprusside anions generated a negative electro-environment in the undulating 1D channels for the preferential discrimination of C_2_H_2_ over CO_2_. Consequently, Cu(bpy)NP exhibited a high 50/50 C_2_H_2_/CO_2_ selectivity and 1/99 C_2_H_2_/C_2_H_4_ selectivity. A considerable balance of separation selectivity and adsorption enthalpy was achieved on Cu(bpy)NP. Dynamic breakthrough experiments revealed the advantageous C_2_H_4_ productivity with 99.9% purity. Modeling studies and in-situ IR measurements indicated the compatible pore environment and strong guest-host interactions for efficient C_2_H_2_ separation. This work encourages the community to design and synthesize Hofmann-type adsorbents for challenging separation tasks.

## Methods

All reagents were purchased from commercial companies and used without further purification. Sodium nitroprusside dihydrate (C_5_FeN_6_Na_2_O·2H_2_O, 99.98%, Aladdin), copper nitrate trihydrate (Cu(NO_3_)_2_·3H_2_O, 99.99%, Aldrich), 4,4’-bipyridine (C_10_H_8_N_2_, 98%, Aladdin), and methanol (CH_4_O, anhydrous, 99.9%, Aladdin) were commercially available and used as supplied without further purification. N_2_ (99.999%), acetylene (C_2_H_2_, 99.99%), CO_2_ (99.99%), ethylene (C_2_H_4_, 99.99%), He (99.999%), and mixed gas-mixtures of C_2_H_2_/CO_2_ (50/50, *v/v*), C_2_H_2_/C_2_H_4_ (1/99, *v/v*), C_2_H_2_/C_2_H_4_ (50/50, *v/v*), and C_2_H_2_/CO_2_/C_2_H_4_ (25/25/50, *v/v/v*) were purchased from Nanchang Guoteng Gas Co., Ltd (China).

### Synthesis of Cu(bpy)NP

Typically, Cu(NO_3_)_2_·3H_2_O (0.25 mmol, 60 mg) and sodium nitroprusside (NP, 0.25 mmol, 75 mg) were dissolved in 3 mL H_2_O and then dripped into a methanol solution (30 mL) of 4,4’-bipyridine (bpy, 0.2 mmol, 35 mg) under vigorous stirring. After reacting at room temperature in the dark for 48 h, cyan precipitates were collected by centrifugation, washed thrice with methanol, and dried overnight in a vacuum oven at 333 K (yield: *ca*. 67% based on NP).

Single-crystals of Cu(bpy)NP were synthesized by slow diffusion of a methanol solution (0.4 mL) of NP (0.003 mmol, 0.89 mg) and bpy (0.002 mmol, 0.35 mg) into an aqueous solution (0.4 mL) of Cu(NO_3_)_2_·3H_2_O (0.003 mmol, 0.72 mg) at room temperature in the dark in a watch glass and kept undisturbed, and 1.2 mL of methanol/H_2_O (1:1) was layered between the top and bottom solutions to slow the reaction rate. Light cyan and square prismatic crystals were formed after 1 week.

### Structure simulations

The unit cell structures (e.g., cell parameters and atomic positions) of Cu(bpy)NP were calculated using the Forcite and Castep modules. The Rietveld refinement, a software package for crystal determination from the XRD pattern, was performed to optimize the lattice parameters iteratively until the *w*R_p_ value converges. The pseudo-Voigt profile function was used for whole profile fitting, and the Berrar-Baldinozzi function was used for asymmetry correction during the refinement processes. Line broadening from crystallite size and lattice strain were both considered.

### Gas adsorption measurements

Equilibrium and kinetic adsorptions of C_2_H_2_, CO_2_, and C_2_H_4_ at 273, 298, and 323 K were measured on Micromeritics ASAP 2460 adsorption apparatus (Micromeritics Instruments, USA). The kinetic adsorptions of C_2_H_2_, CO_2_, and C_2_H_4_ were obtained on Intelligent Gravimetric Analyzer (IGA-100, HIDEN). To remove all the guest solvents in the framework, the fresh powder samples were evacuated under a high vacuum at 373 K for 12 h. The BET surface area was calculated using the adsorption branch with the relative pressure P/P_0_ range of 0.005–0.3. The total pore volume (V_tot_) was calculated based on the adsorbed amount of nitrogen at the P/P_0_ of 0.99. The pore size distribution (PSD) was calculated based on CO_2_ and N_2_ adsorption isotherms at 195 K and 77 K, respectively. The helium gas was used to determine the free space of the system. The sample was degassed for 24 h between each measurement.

### In-situ infrared (IR) spectroscopic measurements

All the IR spectroscopic data are recorded in a Tensor 27 FTIR spectrometer (Bruker, GER) equipped with a liquid N_2_-cooled mercury cadmium telluride MCT-A detector. A vacuum cell, purchased from Specac Ltd., UK (product number P/N 5850c), is placed in the sample compartment of the infrared spectrometer with the sample at the beam’s focal point. The cell is connected to different gas lines (C_2_H_2_, CO_2_, and C_2_H_4_) and a vacuum line for evacuation. The Cu(bpy)NP (powder, ~30 mg) was placed in the cell, and firstly annealed at 100 °C under vacuum for activation and then cooled to RT for recording the reference spectrum. C_2_H_2_ was introduced into the cell, and the spectra were recorded during the gas exposure till 60 min. After fully evacuating the same sample by pumping the cell, the reference spectrum was retaken. Loading of CO_2_ and C_2_H_4_ was performed separately, and the infrared data were recorded in the same manner.

### Breakthrough experiments

The breakthrough experiments were performed on a self-assembly device. Typically, the activated Cu(bpy)NP (about 1.65 g) was packed into a stainless-steel column (4.6 mm inner diameter × 100 mm). The column was first purged with a He flow (10 mL min^−1^) at room temperature for 8 h before breakthrough measurements. The binary C_2_H_2_/CO_2_ (50/50, *v/v*), C_2_H_2_/C_2_H_4_ (1/99, *v/v*), and ternary C_2_H_2_/CO_2_/C_2_H_4_ (25/25/50, *v/v/v*) gas-mixtures were then introduced at a flow rate of 2.0, 3.0, and 5.0 mL min^−1^, respectively. The outlet gas from the column was monitored using gas chromatography (GC-490 plus) with a flame ionization detector. After the breakthrough measurement, the columns packed with samples were regenerated by purging dry He gas (10 mL min^−1^) at 100 °C for 24 h.

### DFT calculations

First-principles DFT calculations were performed using Materials Studio’s CASTEP code. All calculations were conducted under the generalized gradient approximation (GGA) with Perdew-Burke-Ernzerhof (PBE). The optimized structures are in great consistency with the experimentally determined crystal structures. The energy, force, and displacement convergence criteria were set as 1 × 10^−5^ Ha, 2 × 10^−3^ Ha, and 5 × 10^−3^ Å, respectively. Single point energy calculations with the same parameters using Dmol^3^ were performed on optimized Cu(bpy)NP. The electron density data obtained from these calculations were used to construct the 0.015 e^−^ Å^−3^ electron density isosurfaces of the C_2_H_2_ and CO_2_ molecules, while the electron density data of both frameworks were used to construct the 0.15 e^−^ Å^−3^ electron density isosurfaces, with a grid interval of 0.1 Å. The calculated electrostatic potential for Cu(bpy)NP and C_2_H_2_ and CO_2_ molecules were then mapped onto their electron density isosurfaces. A semiempirical addition of dispersive forces to conventional DFT was included in the calculation to account for van der Waals interactions. Cutoff energy of 544 eV and a 2 × 2 × 2 k-point mesh were enough for the total energy coverage within 0.01 meV atom^−1^. The structures of the synthesized materials were first optimized from the reported crystal structures. The pristine structure and an isolated gas molecule placed in a supercell (with the same cell dimensions as the pristine crystal structure) were optimized and relaxed as references to obtain the binding energy. C_2_H_2_, CO_2_, and C_2_H_4_ gas molecules were then introduced to different locations of the channel pore, followed by a full structural relaxation. The static binding energy was calculated by the equation *E*_B_ = *E*(gas) + *E*(adsorbent) − *E*(adsorbent + gas).

### GCMC calculations

All the GCMC simulations were performed in MS 2017R2 package. The crystal structure of the Cu(bpy)NP was chosen after the DFT geometry optimization. The framework and the individual C_2_H_2_, CO_2_, and C_2_H_4_ were considered rigid during the simulation. The charges for atoms of the Cu(bpy)NP and gas components were derived from the Muliken method. The simulations adopted the fixed pressure task, the Metropolis method in the sorption module, and the universal force field (UFF). The interaction energy between the adsorbed molecules and the framework was computed through the Coulomb and Lennard–Jones 6-12 (LJ) potentials. The cutoff radius was chosen 18.5 Å for LJ potential and the electrostatic interactions were handled using the Ewald summation method. The loading and equilibration steps were 1 × 10^7^, and the production steps were 1 × 10^7^.

### Sample characterizations

PXRD was collected on a PANalytical Empyrean Series 2 diffractometer with Cu Kα radiation (*λ* = 1.540598 Å), which operated at 40 kV, 40 mA, and a scan speed of 0.0167°, a scan time of 15 s per step, and 2θ ranging from 5 to 60° at room temperature. The thermogravimetric analysis (TGA) data were performed on a NETZSCH Thermogravimetric Analyzer (STA2500) from 25 to 800 °C with a heating rate of 10 °C/min under an N_2_ atmosphere. The single-component adsorption isotherms of C_2_H_2_, CO_2_, and C_2_H_4_ at 273, 298, and 323 K were measured on Micromeritics ASAP 2460 adsorption apparatus (Micromeritics Instruments, USA). The degassing procedure for all samples was carried out at 373 K under vacuum for 12 h before each adsorption measurement. The specific BET surface area was calculated based on the CO_2_ adsorption isotherm data (0.05 and 0.15 relative pressure) at 195 K. The PSD was derived from the adsorption branch of CO_2_ isotherms using the non-local density functional theory method and assuming a slit pore model.

### Single-crystal X-ray diffraction

Single-crystal X-ray diffraction data for Cu(bpy)NP were collected at **193(2) K** on a Bruker-AXS D8 VENTURE diffractometer equipped with a PHOTON-100/CMOS detector (GaKα, *λ* = 1.3414 Å). Indexing was performed using APEX2. SaintPlus 6.01 was used to complete data integration and reduction. The multi-scan method implemented in SADABS was used to conduct absorption correction. XPREP implemented in APEX2.1 was used to determine the space group. The structures were solved by direct methods and refined by nonlinear least-squares on the F2 method with SHELXL-97 contained in APEX2, OLEX2 v1.1.5, and WinGX v1.70.01 program packages. The Squeeze routine implemented in Platon was used to treat the contribution of disordered solvent molecules as diffuse. Crystallographic data are available free of charge from the Cambridge Crystallographic Data Center (CCDC). The CCDC number for the Cu(bpy)NP crystal is 2124121.

### Structural stability tests

Solvent stability tests were performed by placing 100 mg samples in 20 mL vials containing 15 mL of different organic solvents for 1 week, boiling water for 3 h, and acid/basic solutions with different pH for 1 week. Then, the solid was separated by filtration and subsequently activated at 100 °C for 12 h, and PXRD tests characterized the structure of the materials.

### Fitting of isotherms

The single-component isotherms for C_2_H_2_, CO_2_, and C_2_H_4_ on Cu(bpy)NP were fitted with the dual-site Langmuir-Freundlich isotherm model:

1 $$q={q}_{1}\frac{{b}_{1}{p}^{{v}_{1}}}{1+{b}_{1}{p}^{{v}_{1}}}+{q}_{2}\frac{{b}_{2}{p}^{{v}_{2}}}{1+{b}_{2}{p}^{{v}_{2}}}$$

with T-dependent parameters *b*

2 $$b={b}_{0}{\exp }\left(\frac{E}{{RT}}\right)$$

Where *q* is the adsorbed amount for an adsorbent in mmol g^−1^, *q*_1_, *q*_2_ is the saturated adsorption capacities in mmol g^−1^, *b* is the Langmuir parameters in kPa^−1^, *p* is pressure in kPa^−1^, *ν* is the Freundlich parameters for single-sites, *R* is the gas constant, and *T* (K) is the temperature. The model fits the pure component isotherms well, and the *R*^2^ values are more significant than 0.9994 (see Table S6).

### IAST calculations of adsorption selectivity

IAST calculations of adsorption selectivity for C_2_H_2_/CO_2_ and C_2_H_2_/C_2_H_4_ separation was defined by

3 $${S}_{{ads}}=\frac{{q}_{1}/{q}_{2}}{{p}_{1}/{p}_{2}}$$

q_1_ and *q*_2_ are the molar loadings in the adsorbed phase in equilibrium with the bulk gas phase, *p*_1_ and *p*_2_ are partial pressures.

### Separation factor/separation selectivity calculations

The amount of adsorbed gas *i* (*q*_*i*_) is calculated from the breakthrough curve as follows:

4 $${q}_{i}=\frac{{V}_{i}{T}_{0}-\,{V}_{dead}-\int_{0}^{{t}_{0}}V_{e}\triangle T}{m}$$

Here, *V*_*i*_ is the influent flow rate of gas (cm^3^ min^−1^), *V*_*e*_ is the effluent flow rate of gas (cm^3^ min^−1^), *V*_dead_ is the dead volume of the system (cm^3^), *t* is the adsorption time (min) and *m* is the mass of the sorbent (*g*)^1^.

On approximation, this simplifies to:

5 $${q}_{i}=\frac{{V}_{T}{\triangle {TP}}_{i}}{m}$$

*V*_*T*_ is the total flow rate of gas (cm^3^ min^−1^), *P*_*i*_ is the partial pressure of gas *i* (bar) and *ΔT* is the time for the initial breakthrough of gas *i* to occur (min)^2^. The separation factor, also known as separation selectivity (*α*_AC_) for the breakthrough experiment, i.e., breakthrough-derived selectivity is determined as follows:

6 $$\alpha=\frac{{q}_{1\;{y}_{2}}}{{q}_{2\;{y}_{1}}}$$

*y*_*i*_ is the partial pressure of gas *i* in the gas-mixture. If one gas component has negligible adsorption, the amount of gas adsorbed is treated as ≤1 cm^3^ for calculations.

### Isosteric heat of adsorption

The binding energies of C_2_H_2_, CO_2_, and C_2_H_4_ are reflected indirectly in the isosteric heat of adsorption, *Q*_st_, which are calculated by the Clausius-Clapeyron equation, defined as

7 $${{Q}}_{{{{{{\rm{st}}}}}}}={-}{{{{{{\rm{RT}}}}}}}^{2}\left(\frac{\partial {{{{{\rm{lnP}}}}}}}{\partial {{{{{\rm{lnT}}}}}}}\right){{n}}_{{{{{{\rm{a}}}}}}}$$

where *Q*_st_ represents the adsorption heat of C_2_H_2_, C_2_H_4_, and CO_2_, *P*, and *T* represent the pressure and temperature under adsorption measurement conditions, and *R* is the universal gas constant. Here, the adsorption heat of each component was determined precisely according to the virial fitting parameters of single-component adsorption isotherms measured at 273, 298, and 323 K up to 1.0 bar, which was defined as follows:

8 $${{{{{\rm{InP}}}}}}={{{{{\rm{In}}}}}}\,{{{{{\rm{N}}}}}}+\frac{1}{{T}}\,\mathop{\sum }\limits_{{i}=0}^{{m}}{{{{{{\rm{a}}}}}}}_{{i}}{{{{{{\rm{N}}}}}}}^{{i}}\,+\,\mathop{\sum }\limits_{{i}=0}^{{n}}{{{{{{\rm{b}}}}}}}_{{i}}{{{{{{\rm{N}}}}}}}^{{i}}$$

9 $${{Q}}_{{{{{{\rm{st}}}}}}}=-{{{{{\rm{R}}}}}}\mathop{\sum }\limits_{{i}=0}^{{m}}{{{{{{\rm{a}}}}}}}_{{i}}{{{{{{\rm{N}}}}}}}_{{i}}$$

where the *N* is the adsorption amount, and *m* and *n* determine the number of items required to precisely fit the adsorption isotherms.

## Supplementary information

Supplementary Information

## Data Availability

All data supporting the finding of this study are available within this article and its Supplementary Information. Crystallographic data for the structures in this article have been deposited at the Cambridge Crystallographic Data Center under deposition nos. CCDC 2124121 (Cu(bpy)NP). Copies of the data can be obtained free of charge from www.ccdc.cam.ac.uk/data_request/cif. Source data supporting this study’s findings are available upon request to J.W.
