# Supplementary material for: Negative electrostatic potentials in a Hofmann-type metal-organic framework for efficient acetylene separation
Source: Nat Commun. 2022 Sep 20;13:5515. doi: 10.1038/s41467-022-33271-3 (PMC9489771; doi:10.1038/s41467-022-33271-3)
Supplement: Supplementary file 1 — Supplementary Information [file 41467_2022_33271_MOESM1_ESM.pdf]

Supplementary Information

Negative Electrostatic Potentials in a Hofmann-type Metal-Organic Framework for Efficient Acetylene Separation

Liu et al.

General Information and Procedures

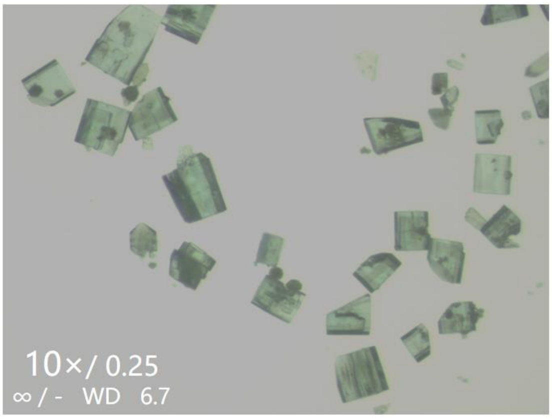

Supplementary Figure 1. Single-crystals. Optical image of as-synthesized Cu(bpy)NP single-crystals.

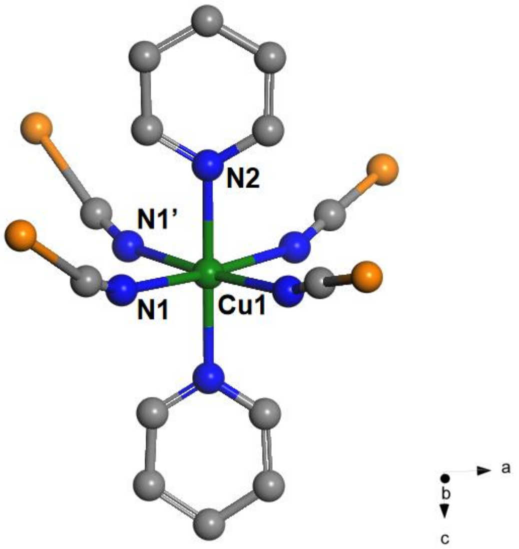

Supplementary Figure 2. The octahedral coordination environment of Cu^{2+} in Cu(bpy)NP. The rest of bpy and NP ligands and hydrogen atoms are omitted for clarity. Green = Cu, grey = C, blue = N, orange = Fe. Selected bond lengths: Cu1-N1 = 2.1940(12) Å, Cu1-N2 = 2.0938(12) Å. Selected bond angles: N1-Cu1-N1 = 180° (extract by symmetry), N1-Cu1-N2 = 87.71(3)°, N1-Cu1-N1' = 89.32(7)°.

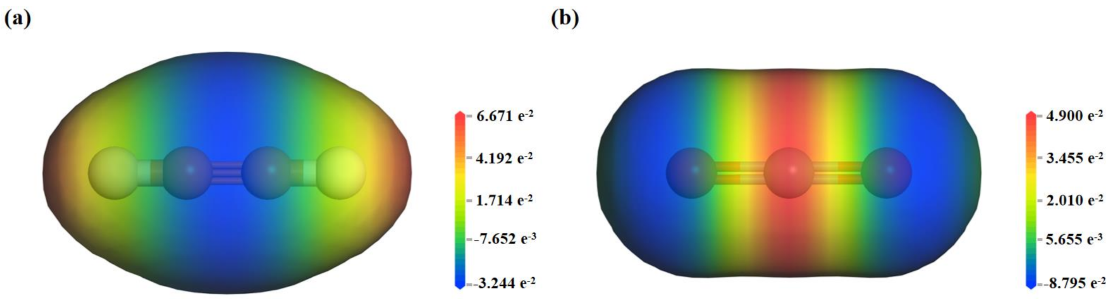

Supplementary Figure 3. Isosurface maps. Isosurface maps of the MEP for (a) C_2H_2 and (b) CO_2 (isovalue = 0.01 au). Red and blue colors represent the positive and negative part of MEP, respectively.

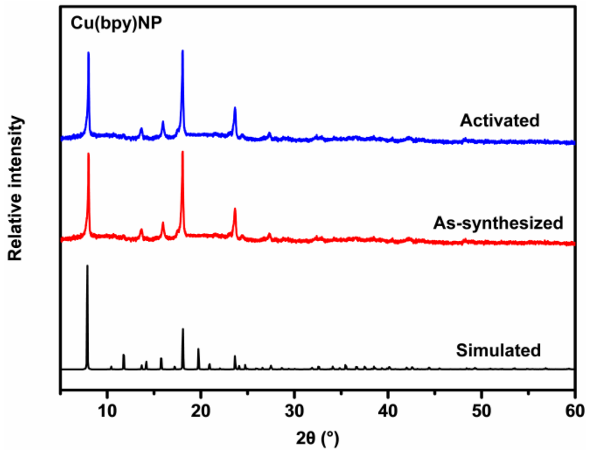

Supplementary Figure 4. PXRD patterns. The powder X-ray diffraction patterns of Cu(bpy)NP under different conditions.

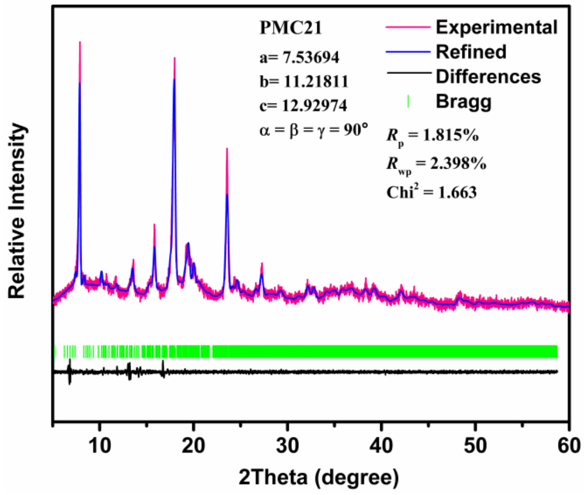

Supplementary Figure 5. PXRD Rietveld refinement. The powder X-ray diffraction Rietveld refinement plot of Cu(bpy)NP.

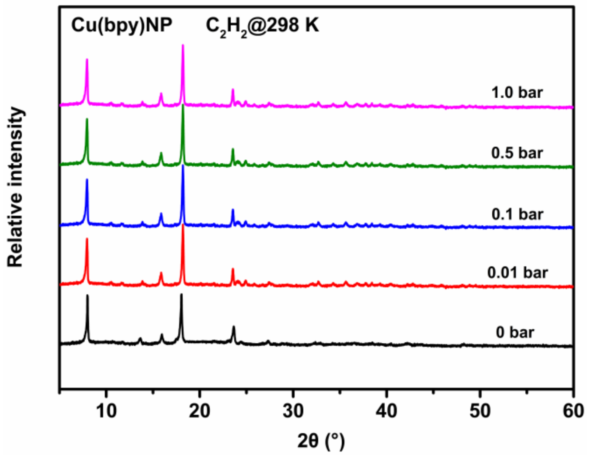

Supplementary Figure 6. PXRD patterns of C_2H_2-loaded samples. The powder X-ray diffraction patterns of C_2H_2-loaded Cu(bpy)NP under different pressures (0-1.0 bar) at 298 K.

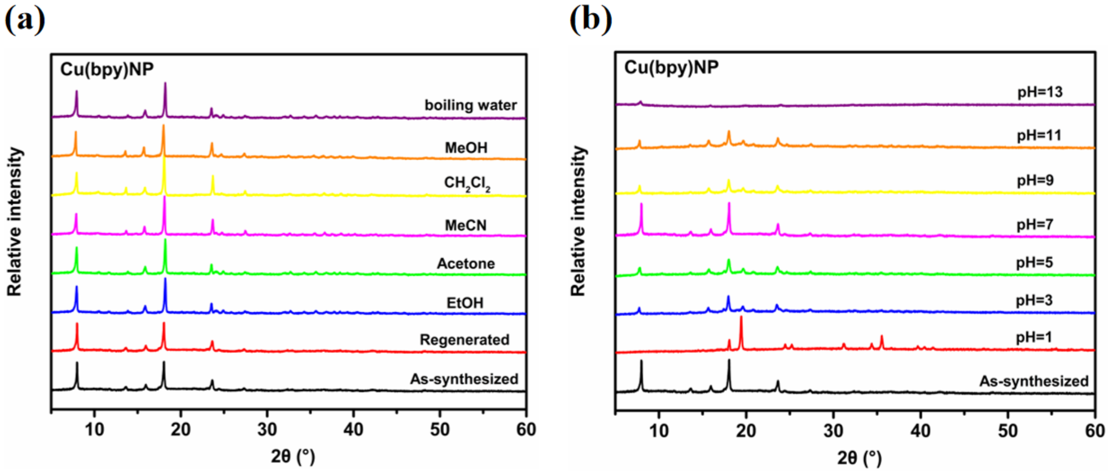

Supplementary Figure 7. PXRD patterns after treatments. The powder X-ray diffraction patterns of Cu(bpy)NP after immersing in (a) different organic solvents for one week; (b) boiling water for 2 h and acid/basic solutions with different pH for one week.

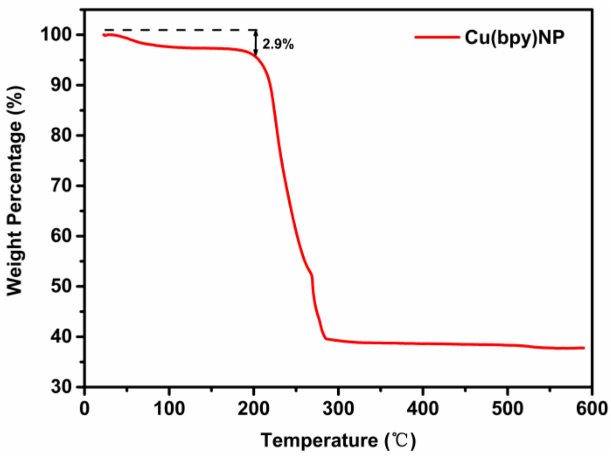

Supplementary Figure 8. TGA curves. TGA curves of the as-synthesized Cu(bpy)NP.

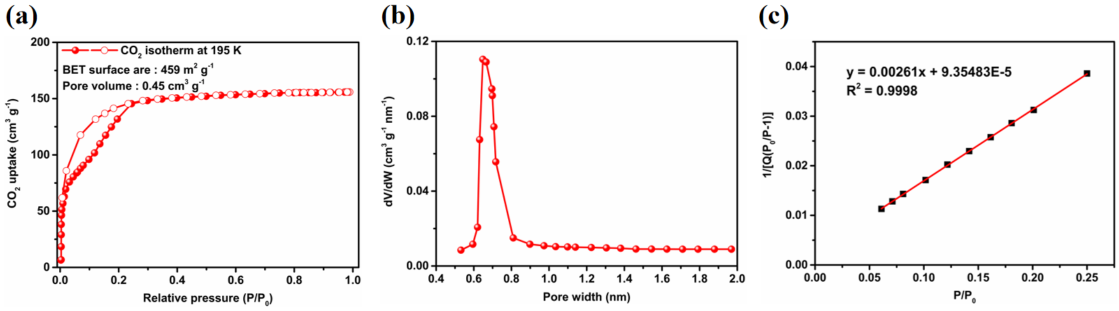

Supplementary Figure 9. CO_2 adsorption isotherms and BET calculation plot. (a) CO_2 adsorption isotherm of Cu(bpy)NP at 195 K; (b) Pore size distribution for Cu(bpy)NP based on Horvath-Kawazoe model determined by CO_2 adsorption isotherm; (c) BET calculation plot for Cu(bpy)NP based on its corresponding CO_2 adsorption isotherm at 195 K.

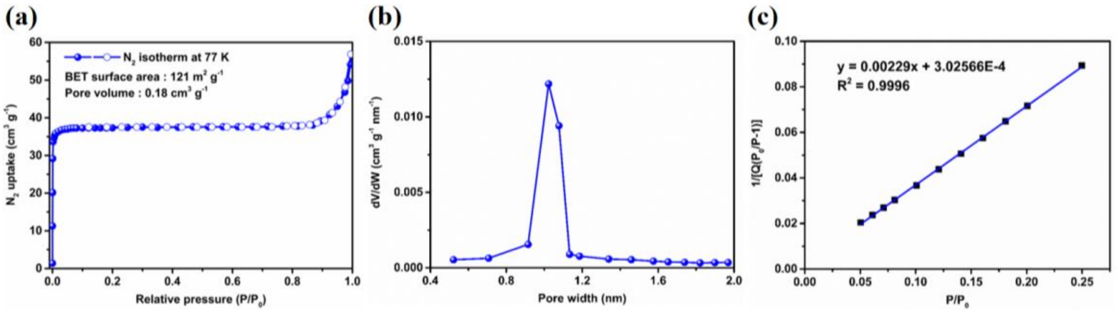

Supplementary Figure 10. N_2 adsorption isotherms and BET calculation plot. (a) N_2 adsorption isotherm of Cu(bpy)NP at 77 K; (b) Pore size distribution for Cu(bpy)NP based on DFT model determined by N_2 adsorption isotherm; (c) BET calculation plot for Cu(bpy)NP based on its corresponding N_2 adsorption isotherm at 77 K.

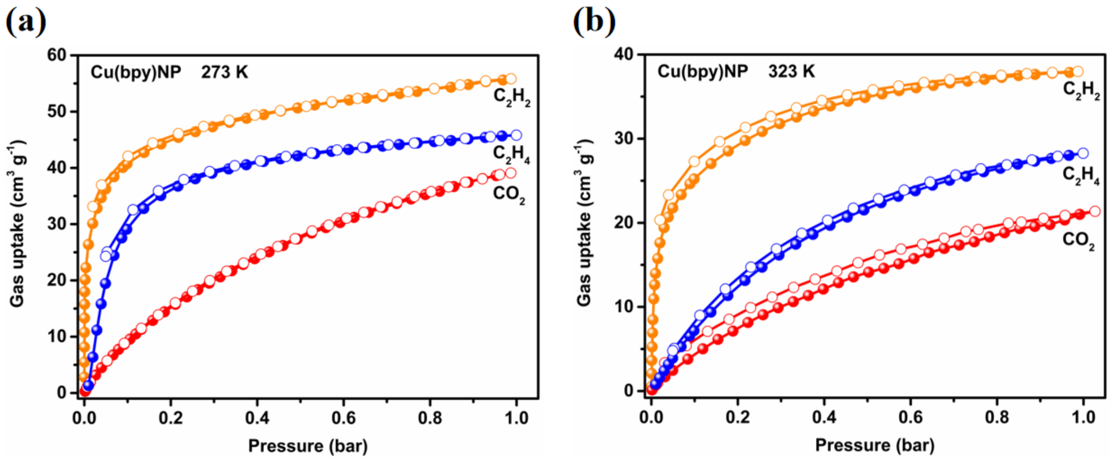

Supplementary Figure 11. C_2H_2, CO_2, and C_2H_4 adsorption isotherms for Cu(bpy)NP at 273 and 323 K. Adsorption isotherms of C_2H_2, CO_2, and C_2H_4 on Cu(bpy)NP at (a) 273 K and (b) 323 K.

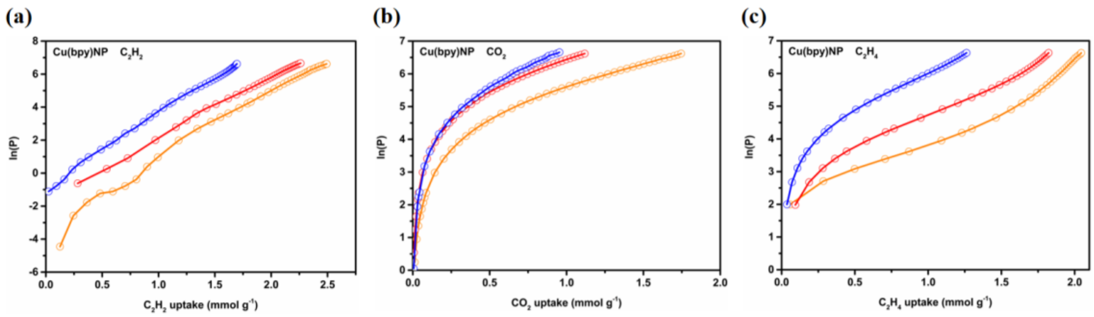

Supplementary Figure 12. The Virial fitting curves of C_2H_2, CO_2, and C_2H_4 isotherms. Virial fitting curves of (a) C_2H_2, (b) CO_2, and (c) C_2H_4 adsorption isotherms on Cu(bpy)NP at 273, 298, and 323 K up to 1.0 bar.

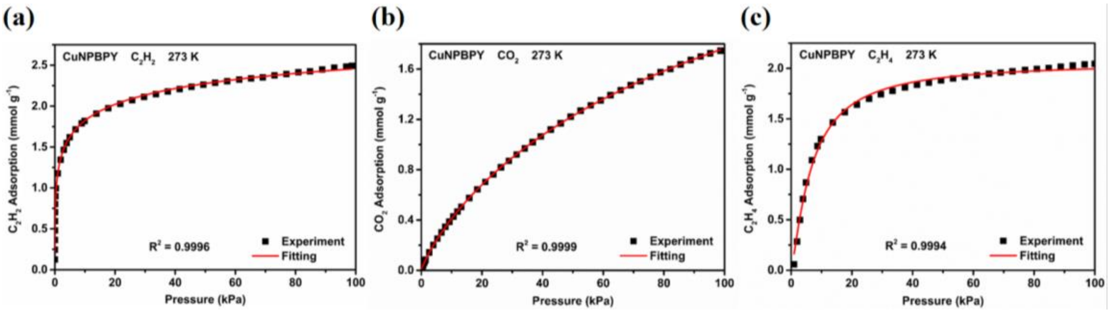

Supplementary Figure 13. The Langmuir-Freundlich fittings of C_2H_2, CO_2, and C_2H_4 isotherms at 273 K. Adsorption isotherms of (a) C_2H_2, (b) CO_2, and (c) C_2H_4 on Cu(bpy)NP at 273 K fitted by the dual-site Langmuir-Freundlich isotherm model.

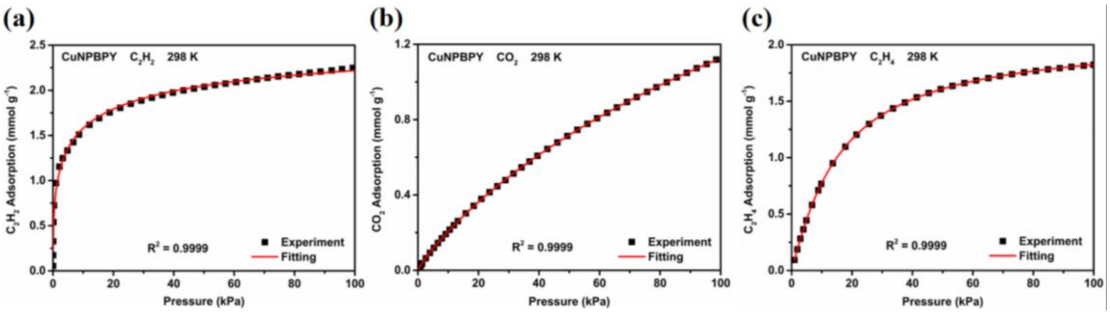

Supplementary Figure 14. The Langmuir-Freundlich fittings of C_2H_2, CO_2, and C_2H_4 isotherms at 298 K. Adsorption isotherms of (a) C_2H_2, (b) CO_2, and (c) C_2H_4 on Cu(bpy)NP at 298 K fitted by the dual-site Langmuir-Freundlich isotherm model.

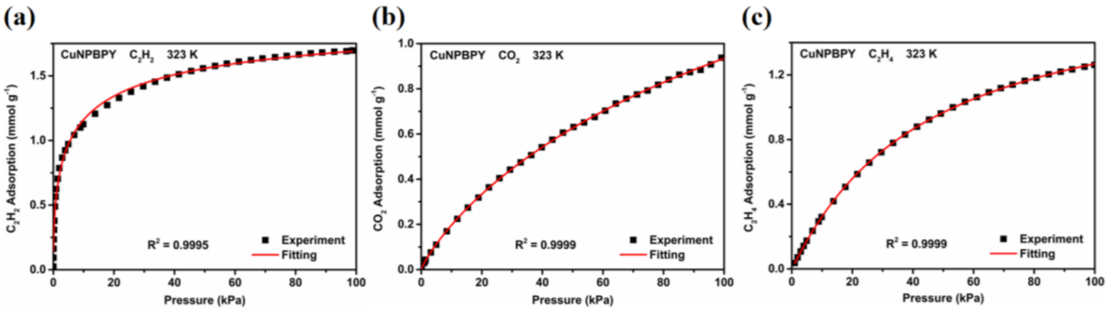

Supplementary Figure 15. The Langmuir-Freundlich fittings of C_2H_2, CO_2, and C_2H_4 isotherms at 323 K. Adsorption isotherms of (a) C_2H_2, (b) CO_2, and (c) C_2H_4 on Cu(bpy)NP at 323 K fitted by the dual-site Langmuir-Freundlich isotherm model.

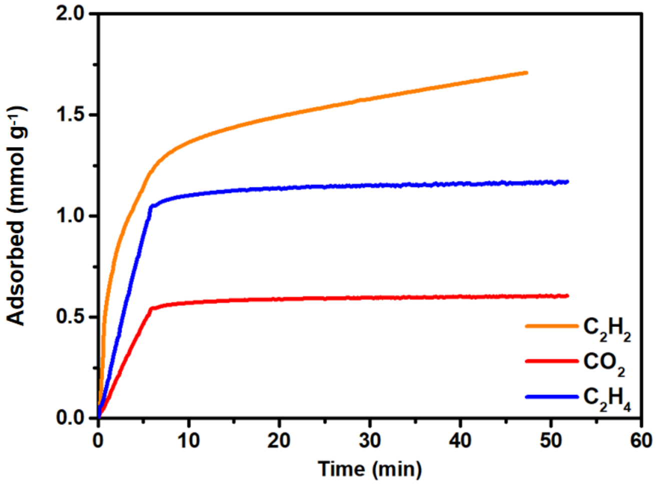

Supplementary Figure 16. C_2H_2, CO_2, and C_2H_4 adsorption kinetic curves for Cu(bpy)NP. Adsorption kinetic profiles of C_2H_2, CO_2, and C_2H_4 on Cu(bpy)NP at 298 K and 0.4 bar.

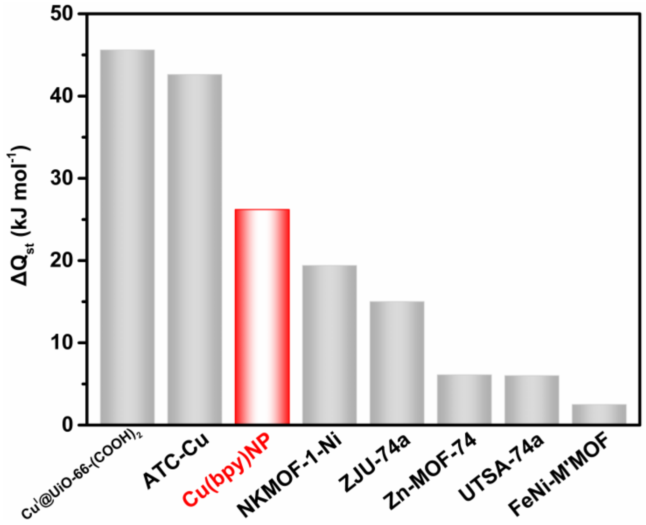

Supplementary Figure 17. Comparison of low-loading ΔQ_{st}. Low-loading ΔQ_{st} (C_2H_2 and CO_2) for leading C_2H_2/CO_2 separation adsorbents with OMSs.

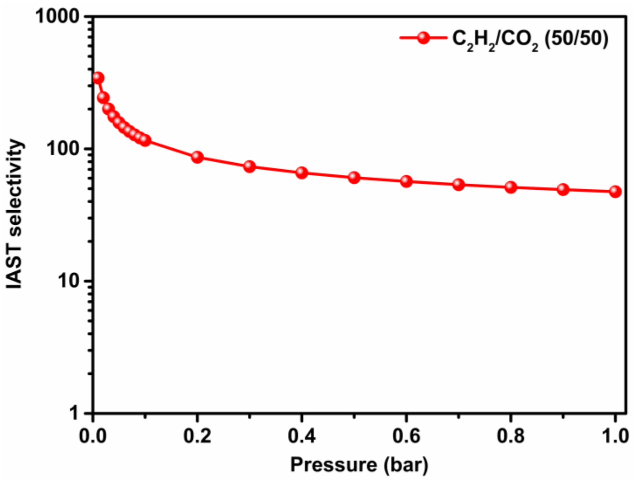

Supplementary Figure 18. 50/50 C_2H_2/CO_2 IAST selectivity plots. The 50/50 C_2H_2/CO_2 selectivity for Cu(bpy)NP at 298 K and 1.0 bar.

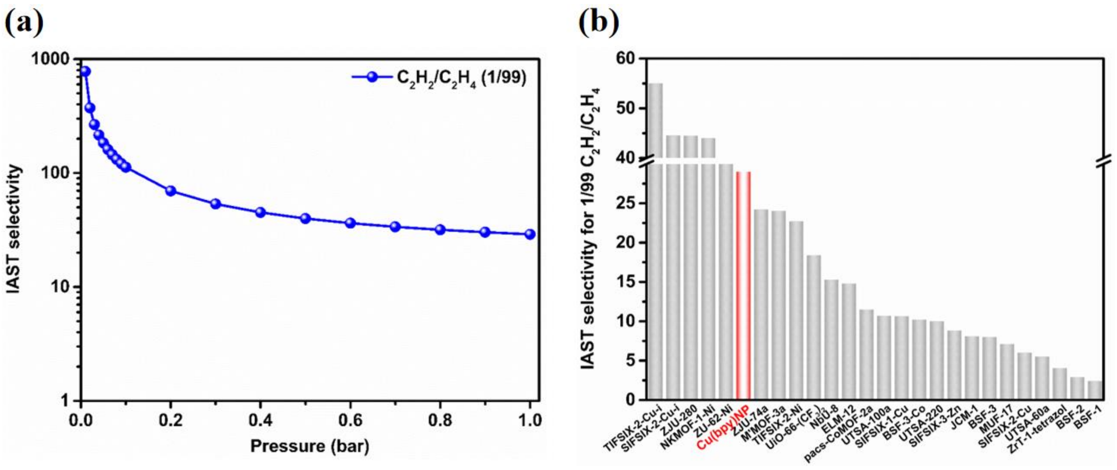

Supplementary Figure 19. 1/99 C_2H_2/C_2H_4 IAST selectivity plots. (a) The 1/99 C_2H_2/C_2H_4 selectivity for Cu(bpy)NP at 298 K and 1.0 bar. (b) Comparison of the 1/99 C_2H_2/C_2H_4 selectivity for Cu(bpy)NP with other best-performing adsorbents at 1.0 bar.

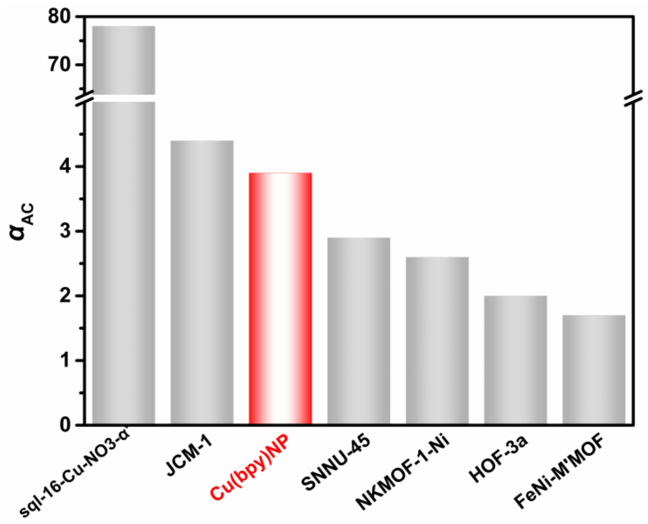

Supplementary Figure 20. Separation selectivity (α_{AC}). Comparison plot of separation selectivity (α_{AC}) for C_2H_2/CO_2 (50/50, v/v) of Cu(bpy)NP based on the breakthrough experiment with other best-performing adsorbents at 298 K.

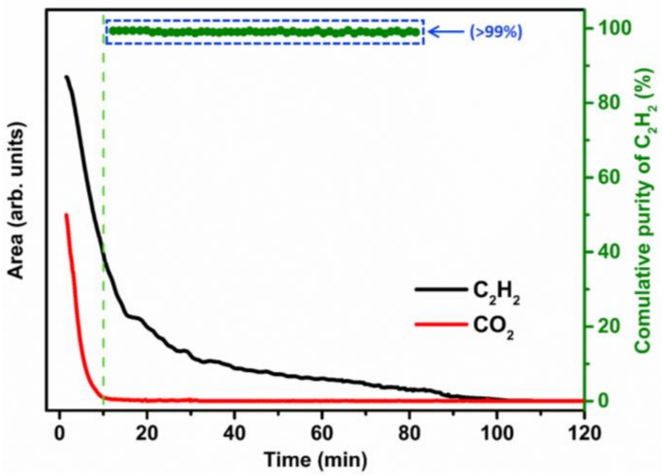

Supplementary Figure 21. Desorption curves for Cu(bpy)NP. The signals of desorbed C_2H_2 and CO_2 during the regeneration process under a He flow rate of 10 mL/min at 298 K.

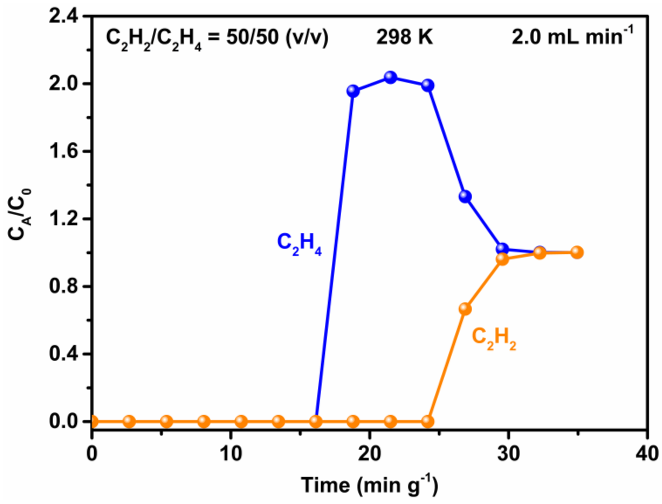

Supplementary Figure 22. Breakthrough curve for Cu(bpy)NP. Breakthrough curve for C_2H_2/C_2H_4 (50/50, *v/v*) gas-mixture on Cu(bpy)NP at 298 K (mixed gas flow rate: 2.0 mL min^{-1}).

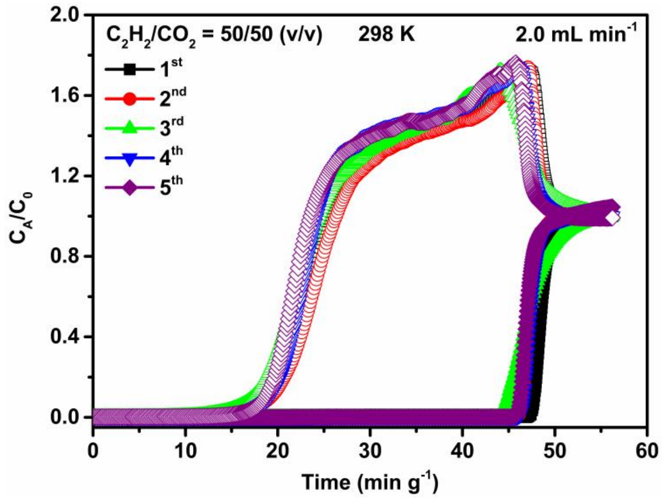

Supplementary Figure 23. Cycling Breakthrough curves. Cycling breakthrough tests for C_2H_2/CO_2 (50/50, *v/v*) on Cu(bpy)NP at 298 K (mixed gas flow rate: 2.0 mL min^{-1}).

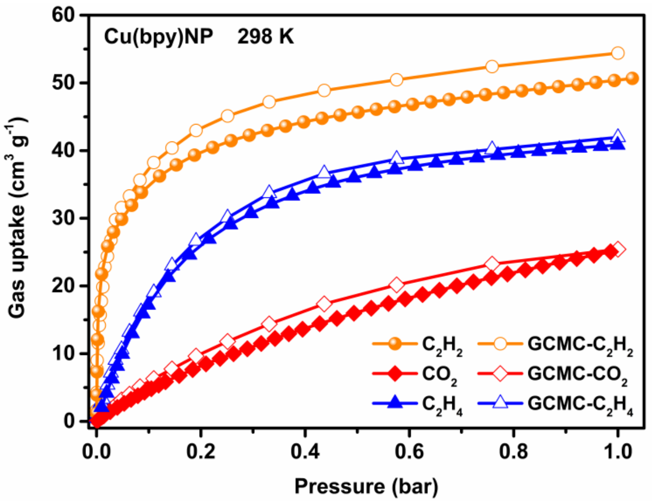

Supplementary Figure 24. Experimental and GCMC-calculated C_2H_2, CO_2, and C_2H_4 adsorption isotherms. Comparison of experimental and GCMC-calculated C_2H_2, CO_2, and C_2H_4 adsorption isotherms at 298 K.

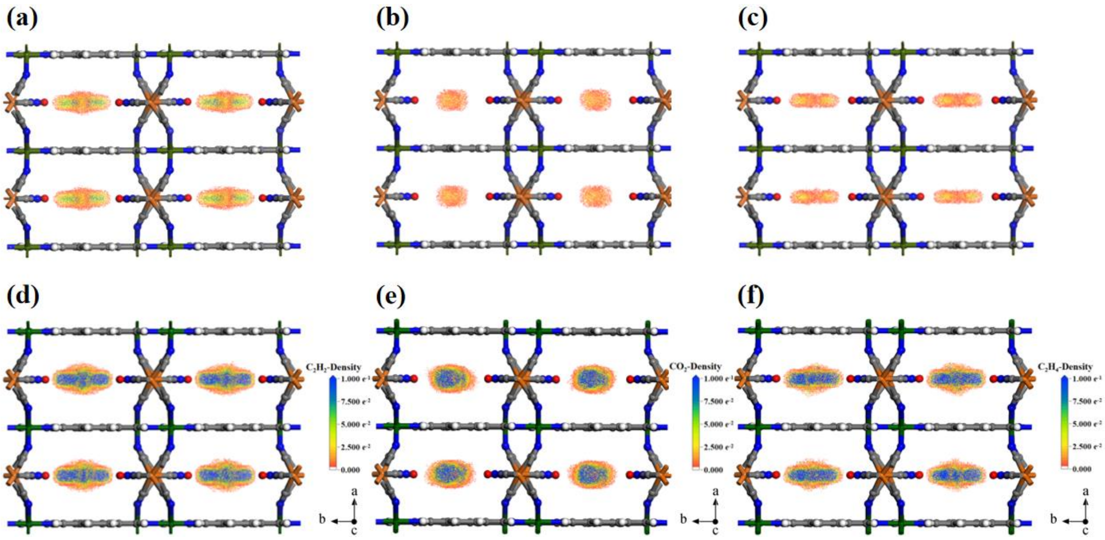

Supplementary Figure 25. Distribution densities of C_2H_2, CO_2, and C_2H_4. GCMC simulations for the distribution density of (a) C_2H_2, (b) CO_2, and (c) C_2H_4 in Cu(bpy)NP at 1 kPa and 298 K; GCMC simulations for the distribution density of (d) C_2H_2, (e) CO_2, and (f) C_2H_4 in Cu(bpy)NP at 100 kPa and 298 K.

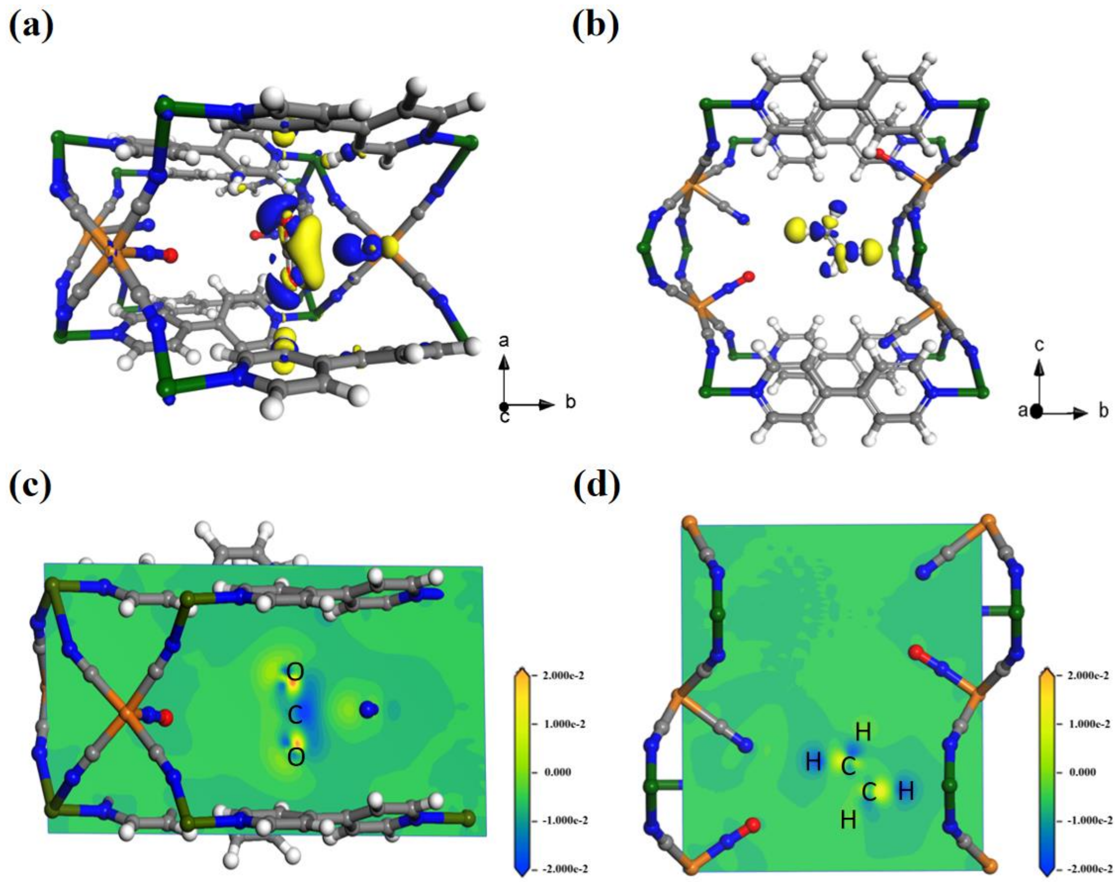

Supplementary Figure 26. Charge density difference plots. Charge density difference plots showing the interaction between (a,c) CO_2, (b,d) C_2H_4 molecules and Cu(bpy)NP.

Supplementary Table 1. Comparison of molecular sizes and physical properties of C_2H_2, CO_2, and C_2H_4.

Compounds	C_2H_2	CO_2	C_2H_4
Molecule model	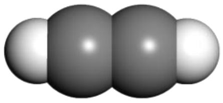	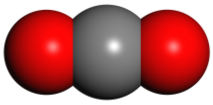	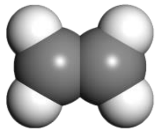
Size (Å^3)	3.32 × 3.34 × 5.70	3.18 × 3.33 × 5.36	3.28 × 4.18 × 4.84
Kinetic diameter (Å)	3.3	3.3	4.2
Boiling point (K)	189.3	194.7	169.5
Quadrupole moment (esu cm^2)	7.2×10^{-26}	4.3×10^{-26}	1.5×10^{-26}
Polarizability (Å^3)	3.59	2.65	4.25

Supplementary Table 2. Lattice parameters of the modeled structure of as-synthesized Cu(bpy)NP.

Unit cell parameters	Cu(bpy)NP
Formula	$C_{30}H_{16}N_{16}O_2Fe_2Cu_2$
Formula weight	870.78
Crystal system	Orthorhombic
Space group	*Pmma*
a (Å)	12.9412
b (Å)	7.5154
c (Å)	11.2201
α (°)	90.000
β (°)	90.000
γ (°)	90.000
V (Å^3)	1091.23
Z	1.000
D_{calcd} (g cm^{-3})	1.32595
R_p[a]	0.0262
R_{wp}[b]	0.0365
GOF	1.63
CCDC No.	2124121

$a$$R_p = \sum|cY_{sim}(2\theta i) - I_{exp}(2\theta i) + Y_{back}(2\theta i)|/\sum|I_{exp}(2\theta i)|$.

$b$$R_p = \{w_p[cY_{sim}(2\theta i) - I_{exp}(2\theta i) + Y_{back}(2\theta i)]_2/\sum w_p[I_{exp}(2\theta i)]_2\}_{1/2}$, $w_p = 1/I_{exp}(2\theta i)$.

Supplementary Table 3. PXRD Rietveld refinement plot of the modeled structure of activated Cu(bpy)NP.

Unit cell parameters	Cu(bpy)NP
Formula	$C_{30}H_{16}N_{16}O_2Fe_2Cu_2$
Formula weight	1093.21
Crystal system	Orthorhombic
Space group	*PMC21*
a (Å)	12.92974
b (Å)	7.53694
c (Å)	11.21811
α (°)	90.000
β (°)	90.000
γ (°)	90.000
V (Å^3)	1091.23
Z	1.000
D_{calcd} (g cm^{-3})	1.32355
R_p[a]	0.01815
R_{wp}[b]	0.02398
GOF	1.663

aR$_p$ = $\sum|cY_{sim}(2\theta i)$ - $I_{exp}(2\theta i)$ + $Y_{back}(2\theta i)|/\sum|I_{exp}(2\theta i)|$.

bR$_p$ = $\{w_p[cY_{sim}(2\theta i)$ - $I_{exp}(2\theta i)$ + $Y_{back}(2\theta i)]2/\sum w_p[I_{exp}(2\theta i)]2\}1/2$, $w_p = 1/I_{exp}(2\theta i)$.

Supplementary Table 4. List of atomic coordinates for the modeled structure of Cu(bpy)NP.

Atoms	*x*	*y*	*z*	*s.o.f*
N2	0.62960	0.79480	0.11430	1.00
C	0.58110	0.68400	0.07860	1.00
Cu01	0.75000	1.00000	0.12211	1.00
N003	0.75000	1.00000	0.30310	1.00
N004	0.75000	1.00000	-0.06450	1.00
C005	0.75000	1.00000	-0.31430	1.00
C006	0.75000	1.00000	-0.44370	1.00
C007	0.83740	1.00000	-0.12560	1.00
H007	0.89950	1.00000	-0.08390	1.00
C008	0.84060	1.00000	-0.25150	1.00
H008	0.90370	1.00000	-0.29120	1.00
C00B	0.83130	1.00000	0.36460	1.00
H00B	0.89370	1.00000	0.32350	1.00
C1	0.83650	1.00000	-0.51100	1.00
H1	0.90070	1.00000	-0.47380	1.00
Fe2	0.50910	0.50000	0.03360	0.50
N4	0.42480	0.50000	0.14500	0.50
O1	0.37500	0.50000	0.21800	0.50
C3	0.38900	0.50000	0.09100	0.50
N3	0.32700	0.50000	0.16400	0.50

Supplementary Table 5. List of atomic coordinates for the modeled structure of Cu(bpy)NP.

Atoms	*x*	*y*	*z*	*s.o.f*
N1	0.7985	-0.1155	-0.1346	1.000
C2	0.6831	-0.0793	-0.0805	1.000
N3	0.7968	0.1139	0.1269	1.000
C4	0.6809	0.0653	0.0811	1.000
Cu17	1.0000	0.1168	0.2471	1.000
N18	1.0000	0.2994	0.2471	1.000
N19	1.0000	0.9355	0.2474	1.000
C20	1.0000	0.6837	0.2477	1.000
C21	1.0000	0.5512	0.2475	1.000
C27	1.0000	0.8739	0.1579	1.000
H28	1.0000	0.9263	0.0871	1.000
C29	1.0000	0.7504	0.1557	1.000
H30	1.0000	0.7082	0.0800	1.000
C31	1.0000	0.3610	0.1580	1.000
H32	1.0000	0.3067	0.0833	1.000
C33	1.0000	0.4847	0.1556	1.000
H34	1.0000	0.5286	0.0806	1.000
C35	1.0000	0.8741	0.3368	1.000
H36	1.0000	0.9264	0.4077	1.000
C37	1.0000	0.7504	0.3393	1.000
H38	1.0000	0.7088	0.4152	1.000
C39	1.0000	0.3608	0.3364	1.000
H40	1.0000	0.3063	0.4060	1.000
C41	1.0000	0.4845	0.3394	1.000
H42	1.0000	0.5284	0.4144	1.000
Fe59	0.5000	-0.0216	0.0090	1.000

N60	0.5000	-0.1450	0.0809	1.000
O61	0.5000	-0.2393	0.1218	1.000
C62	0.5000	0.1085	-0.0875	1.000
N63	0.5000	0.1873	-0.1484	1.000

Supplementary Table 6. List of the raw adsorption isotherm data at 273 K up to 0-1.0 bar.

C_2H_2 (cm^3 g^{-1})		CO_2 (cm^3 g^{-1})		C_2H_4 (cm^3 g^{-1})	
1.53142E-5	2.80939	0.00168	0.28444	0.00968	1.30797
1.00022E-4	5.47678	0.0034	0.54929	0.01975	6.36755
2.23471E-4	8.15436	0.00511	0.80579	0.02875	11.15891
3.84286E-4	10.7761	0.00682	1.05752	0.03892	15.81859
4.32741E-4	13.31168	0.00853	1.30142	0.04891	19.46041
6.00721E-4	15.75375	0.01035	1.51865	0.06842	24.42479
8.97447E-4	17.98361	0.01218	1.72733	0.08657	27.5567
0.00194	20.1231	0.014	1.92935	0.09814	29.10335
0.00344	22.26297	0.02589	3.18365	0.13742	32.77897
0.00953	26.38533	0.03972	4.50548	0.17679	35.07633
0.01927	30.11677	0.05293	5.66885	0.21658	36.75188
0.0296	32.83036	0.06614	6.74254	0.25681	38.06909
0.03907	34.70371	0.07925	7.75032	0.29554	39.05167
0.04943	36.26552	0.09218	8.65198	0.33396	39.81554
0.06873	38.42519	0.10553	9.57048	0.37482	40.55839
0.08899	40.07841	0.11889	10.47092	0.41449	41.14759
0.09873	40.74011	0.13174	11.30333	0.45367	41.61289
0.13803	42.73975	0.15811	12.89032	0.49368	42.12593
0.17748	44.23266	0.185	14.42449	0.53327	42.5497
0.21746	45.42193	0.21072	15.75555	0.57217	42.93493
0.25698	46.42516	0.23718	17.07372	0.61163	43.25732
0.29651	47.31097	0.26262	18.35515	0.65152	43.59153
0.33599	48.08752	0.28877	19.50964	0.69	43.86806
0.37531	48.8088	0.31449	20.62918	0.72888	44.15464
0.41471	49.46994	0.34114	21.72042	0.77033	44.42696

0.45419	50.09035	0.36745	22.82957	0.80942	44.66656
0.49355	50.64884	0.39406	23.8506	0.84841	44.85883
0.53266	51.18422	0.42721	25.07398	0.88773	45.14595
0.57259	51.61165	0.45963	26.19804	0.92745	45.41162
0.61289	52.04822	0.49243	27.36681	0.96632	45.67598
0.65216	52.3905	0.52561	28.42219	0.99954	45.81369
0.69109	52.66593	0.55875	29.39025		
0.73013	53.2142	0.5918	30.28591		
0.76963	53.58045	0.62383	31.20438		
0.80936	54.07456	0.65744	32.09309		
0.84816	54.32515	0.69105	32.96877		
0.88847	54.81427	0.72256	33.6646		
0.92862	55.31713	0.75581	34.47017		
0.96852	55.60967	0.78896	35.27057		
0.98694	55.8338	0.82194	35.84596		
		0.85484	36.67856		
		0.88834	37.45127		
		0.92115	38.09316		
		0.9527	38.62987		
		0.98653	39.10375		

Supplementary Table 7. List of the raw adsorption isotherm data at 298 K up to 0-1.0 bar.

C_2H_2 (cm^3 g^{-1}) 0-1.0 bar		CO_2 (cm^3 g^{-1}) 0-1.0 bar		C_2H_4 (cm^3 g^{-1}) 0-1.0 bar	
2.33E-04	1.21168	2.26E-03	0.16611	9.54E-03	2.08986
4.76E-04	3.87678	4.54E-03	0.32766	1.92E-02	4.19548
8.06E-04	7.30577	6.81E-03	0.48649	2.93E-02	6.29324
0.0017	12.09246	0.00919	0.60627	0.03927	8.16194
0.00325	16.24147	0.01155	0.72548	0.04916	9.94297
0.00976	21.76592	0.01398	0.84092	0.0678	12.98266
0.02135	25.86863	0.02614	1.43576	0.0885	15.90934
0.03244	27.96046	0.03966	2.07423	0.09852	17.19806
0.04792	29.85468	0.05291	2.66885	0.13679	21.28077
0.06705	31.92671	0.06612	3.2463	0.17805	24.58694
0.08615	33.81965	0.07911	3.79263	0.21524	26.94677
0.12077	36.24818	0.0919	4.30998	0.2564	29.0632
0.15276	37.88444	0.10497	4.8246	0.29574	30.72999
0.18771	39.31618	0.11891	5.35879	0.33589	32.15856
0.22271	40.46313	0.13215	5.85403	0.37521	33.33673
0.25777	41.46232	0.15833	6.7752	0.41463	34.34982
0.29278	42.26437	0.18417	7.6563	0.4539	35.2259
0.32765	42.97034	0.20974	8.48912	0.49336	35.97975
0.36256	43.61508	0.23664	9.28659	0.53266	36.63486
0.39731	44.22993	0.26246	9.99519	0.57192	37.1987
0.43241	44.7716	0.28909	10.72288	0.61115	37.7136
0.46704	45.23263	0.31577	11.50586	0.6514	38.18626
0.50163	45.66378	0.34229	12.24143	0.69004	38.59871
0.5365	46.10615	0.36878	12.94111	0.72975	38.95519
0.57144	46.47907	0.39501	13.61476	0.76968	39.31912

0.60627	46.83518	0.42782	14.43313	0.80869	39.62446
0.64114	47.1887	0.46082	15.21097	0.84881	39.90327
0.67607	47.55006	0.49367	15.96835	0.88744	40.15727
0.71092	47.90506	0.52682	16.70989	0.92614	40.41022
0.74594	48.25893	0.55988	17.41666	0.96703	40.6493
0.78075	48.55639	0.59184	18.08155	0.99925	40.82528
0.81569	48.83942	0.62505	18.73562		
0.85051	49.1642	0.65752	19.369		
0.886	49.44266	0.69109	20.02282		
0.92329	49.73601	0.72284	20.60456		
0.95765	50.06011	0.75723	21.22125		
0.99309	50.35104	0.78804	21.77127		
1.02761	50.6565	0.82226	22.36867		
		0.85552	22.9445		
		0.8883	23.48784		
		0.92098	24.03824		
		0.95378	24.53316		

Supplementary Table 8. List of the raw adsorption isotherm data at 323 K up to 0-1.0 bar.

C_2H_2 (cm^3 g^{-1}) 0-1.0 bar		CO_2 (cm^3 g^{-1}) 0-1.0 bar		C_2H_4 (cm^3 g^{-1}) 0-1.0 bar	
4.33E-04	0.54368	1.38E-03	0.12987	9.70E-03	0.8122
6.01E-04	2.08975	9.20E-03	0.65152	1.91E-02	1.59668
8.97E-04	3.64761	1.24E-02	0.8316	2.95E-02	2.42198
0.00164	5.25817	0.0143	0.98096	0.03934	3.17668
0.00253	6.91812	0.03159	1.69764	0.04912	3.90594
0.00346	8.48325	0.04933	2.46309	0.06863	5.2749
0.00549	10.95636	0.08431	3.79842	0.0884	6.56569
0.00745	12.63672	0.11906	5.02579	0.09851	7.18856
0.00954	13.97675	0.15383	6.14126	0.13764	9.38546
0.01458	15.75284	0.18874	7.13254	0.17681	11.35693
0.01981	17.61854	0.22334	8.14916	0.21648	13.13844
0.02936	19.42045	0.25823	9.06182	0.25579	14.72918
0.03904	20.69121	0.29316	9.90097	0.29534	16.1725
0.04934	21.77015	0.32832	10.62767	0.33422	17.46009
0.06889	23.35563	0.36324	11.35076	0.3738	18.62786
0.08906	24.64368	0.39797	12.1259	0.41338	19.70425
0.09882	25.21695	0.43304	12.87531	0.45319	20.68504
0.13786	27.03674	0.46785	13.5728	0.49175	21.52562
0.17774	28.53254	0.5028	14.13225	0.53167	22.39265
0.21691	29.78344	0.53778	14.58346	0.5713	23.15412
0.25634	30.85253	0.57255	15.12751	0.61086	23.81244
0.29685	31.78178	0.60754	15.74615	0.65047	24.47902
0.33596	32.57763	0.64235	16.4513	0.69002	25.05851
0.37531	33.2763	0.67745	16.94637	0.72873	25.53644

0.41438	33.87919	0.71246	17.35593	0.76901	26.06505
0.45486	34.43919	0.74737	17.7552	0.80766	26.48271
0.49434	34.90123	0.78223	18.3179	0.84776	26.96811
0.5339	35.31913	0.81721	18.83063	0.88728	27.33028
0.57294	35.7121	0.85219	19.31883	0.92534	27.65057
0.61255	36.05149	0.88723	19.57377	0.96632	28.01631
0.65247	36.33888	0.92219	19.79525	0.99908	28.26981
0.69078	36.61508	0.95636	20.33778		
0.73189	36.83219	0.9912	20.99947		
0.77065	37.05174	1.02652	21.37075		
0.81058	37.28649				
0.84995	37.50185				
0.88774	37.64418				
0.92757	37.78367				
0.96774	37.86674				
0.98707	38.00042				

Supplementary Table 9. Comparison of the adsorption capacity and C_2H_2/CO_2 (50/50, v/v), C_2H_2/CO_2 (1/99, v/v) selectivity and heat of adsorption data of Cu(bpy)NP with other best-performing materials at 298 K and 1.0 bar.

Sample	S_{BET} (m^2 g^{-1})	Pore size (Å)	C_2H_2 (cm^3 g^{-1}) 0.01 bar/ 1.0 bar	CO_2 (cm^3 g^{-1}) 1.0 bar	C_2H_4 (cm^3 g^{-1}) 1.0 bar	Q_{st}^b (kJ mol^{-1}) C_2H_2	Q_{st}^b (kJ mol^{-1}) CO_2	C_2H_2/CO_2^a (50/50) 1.0 bar	$C_2H_2/C_2H_4^a$ (1/99) 1.0 bar	*Ref.*
**Cu(bpy)NP**	**459**	**6.6**	**22.4/50.7**	**25.1**	**40.8**	**40.8**	**14.6**	**47.2**	**28.5**	**This work**
SIFSIX-3-Ni	223	5.3	4.5/73.9	60.5	-	36.7	50.9	7.7	-	[2]
TIFSIX-2-Cu-i	685	5.2	39.9/91.8	96.3	56	46.3	35.8	10.7	55.0	[2]
UTSA-74a	830	8.0	19.2/103.5	67.9	-	31.0	25.0	9.0	-	[3]
SNNU-45	1006	4.5,5.1	19.7/134.0	97	-	39.9	27.0	8.5	-	[4]
FeNi-M'MOF	383	4.0	29.1/96.1	60.9	-	27.0	24.5	24.0	-	[5]
Cu^I@UiO-66 -$(COOH)_2$	302	4.4,5.0	20.2/51.7	19.0	-	74.5	28.9	185.0	-	[6]
CPL-1-NH_2	103	3.8×4.4	-/41.2	4.7	-	50.0	32.4	119.0	-	[7]
ZNU-1	532	6.9	27.3/76.2	38.1	-	54.0	44.0	56.6	-	[8]

ATC-Cu	600	3.5,4.4	56.9/112.2	90.0	-	79.1	36.5	53.6	-	[9]
ZJU-74a	694	3.6	49/85.8	70.1	71.0	45.0	30.0	36.5	24.2	[10]
NKMOF-1-Ni	382	5.8	39/60.9	51.1	47.3	60.3	40.9	22.0	44.0	[11]
HOF-3a	165	7.0	4.6/47	21	-	19.5	42.0	21.5	-	[12]
DICRO-4-Ni-i	398	6.2,6.6	9.7/43	23	-	37.7	33.9	13.9	-	[13]
JCM-1	550	3.9	10.1/76.6	38.1	35.6	36.9	33.4	13.7	8.1	[14]
FJU-90	1572	5.1×5.4	7.7/180	103	-	25.2	20.7	4.3	-	[15]
JNU-1	818	12.3× 18.2	5.5/27.4	4.1	-	13.0	23.8	6.6	-	[16]
Zn-MOF-74	996	13.6	14.8/124	120	-	24.7	18.6	2.8	-	[17]
ZJU-280	257	4.2×5.5, 3.4×7.5	34.0/92.1	71.0	66.1	50.6	39.0	18.1	44.5	[18]
BSF-1	535	8.4-8.7	3.5/52.6	39.6	36.5	30.7	22.0	3.4	2.4	[19]
BSF-2	403	7.4-7.9	6.7/41.4	29.8	29.6	37.3	28.7	5.1	2.9	[20]
BSF-3	458	6.2-6.8	15.2/80.4	42.3	53.1	42.7	25.5	16.3	8.0	[21]
BSF-3-Co	437	6.2-6.8	15.7/86.2	54.0	56.2	44.3	26.5	12.7	10.2	[21]
pacs-CoMOF-2a	196	5.8,6.6	9.5/121.0	66.1	62.9	34.2	24.0	13.0	11.5	[22]

SIFSIX-1-Cu	1178	8.0	11.8/190.4	108.0	92.1	37.0	26.5	-	10.6	[23]
SIFSIX-2-Cu-i	503	5.2	39.5/90	108.4	49.1	41.9	31.9	-	44.5	[24]
SIFSIX-3-Zn	250	4.2	17.8/81.5	56.9	50.2	31.0	45.0	-	8.8	[24]
TIFSIX-2-Ni-i	480	4.6	17.9/94.3	101.7	54.2	40.0	34.0	6.2[c]	22.7	[25]
ELM-12	706	4.3	6.5/57.3	-	22.4	25.4	-	-	14.8	[26]
UTSA-60a	484	3.6	11.5/70.1	-	45.9	36.0	-	-	5.5	[27]
UTSA-100a	970	4.3	9.6/95.6	-	37.2	22.0	-	-	10.7	[28]
UTSA-220	577	8.8	7.3/76.2	75.7	56.7	29.0	27.0	4.4	10.0	[29]
UTSA-300a	311	3.3	-/69.0	3.36	0.90	57.6	-	-	-	[30]
UiO-66-$(CF_3)_2$	330	6.8	6.2/119.8	23.3	26.9	43.0	20.4	16.0	18.4	[31]
NbU-8	1467	4.5	4.8/190.0	56.9	114.9	34.6	30.3	5.4	15.3	[32]
MUF-17	211	4.7	13.0/67.4	56.2	48.2	49.5	33.8	6.0	7.1	[33]
M'MOF-3a	110	3.4	4.2/42.6	-	9.0	27.1	40.5	8.4	24.0	[34]
ZU-62-Ni	585	3.0-3.9	9.0/67.2	-	17.9	43.0	-	-	37.2	[35]
ZrT-1-tetrazol	637	7.1	4.8/57.8	35.2	28.2	33.3	29.9	2.8	4.1	[36]
SOFOUR-1-Zn	612	4.0	36.9/69	81	-	57.0	33.0	6.6	-	[37]
SIFSIX-22-Zn	641	4.1	17.5/127	95	-	36.5	25.0	6.5	-	[37]

[a] A total pressure of 298 K and 1.0 bar.

[b] At low loading.

[c] IAST selectivity for C_2H_2/CO_2 (2/1, v/v) gas mixtures.

Supplementary Table 10. The virial parameters for calculated Q_{st} of C_2H_2, CO_2, and C_2H_4 on Cu(bpy)NP at 273 K, 298 K, and 323 K up to 1.0 bar.

Viral Coefficient	C_2H_2 Value	CO_2 Value	C_2H_4 Value
a_0	-4907.603	-1759.116	-3827.008
a_1	-114.5948	30.02568	-15.34480
a_2	5.792407	-1.812762	-1.196907
a_3	-0.09668063	0.03733443	0.02243202
a_4	8.482225E-4	-4.718816E-4	-5.293246E-4
a_5	-2.256726E-6	2.238091E-6	5.047769E-6
b_0	14.05816	7.312612	13.80020
b_1	0.2324380	-0.01363226	0.06894118
b_2	-0.004629982	0.001608189	0.002872585
Equation	$y = Ln(x)+1/T(a_0+a_1x+a_2x^2+a_3x^3+a_4x^4+a_5x^5) + (b_0+b_1x+b_2x^2)$		

Supplementary Table 11. The fitting parameters of the dual-site Langmuir-Freundlich equation model for C_2H_2, CO_2, and C_2H_4 adsorption on Cu(bpy)NP.

Cu(bpy)NP								
Temperature (K)	Gas	Adsorbed amount $q_{sat,A}$ (mmol g^{-1})	Equilibrium constant b_A (kPa^{-1})	v_A	Adsorbed amount $q_{sat,B}$ (mmol g^{-1})	Equilibrium constant b_B (kPa^{-1})	v_B	R^2
273		3.623902	0.3217282	0.3258158	0.3334768	159348.2	3.705365	0.9996
298	C_2H_2	2.163179	0.1766314	0.5772234	0.686818	24.13897	1.568327	0.9999
323		1.003528	0.03250151	1.054286	0.8964061	2.055062	1.020645	0.9995
		3.413609	0.01356169	0.930628	0.06857584	0.388586	1.090911	0.9999
298	CO_2	3.670406	-0.008457937	0.8612271	-0.00576503	1.213621	1.317968	0.9999
323		1.539479	0.007936942	1.110465	0.04880369	1.255334	1.152691	0.9999
273		6.75108	1.371912	0.6131056	-4.440349	6.514081	2.19025	0.9994
298	C_2H_4	2.024209	0.04463897	1.121365	0.02991056	0.6351896	0.6999196	0.9999
323		1.724995	0.01730339	1.087233	0.02299416	1.032653	0.9998291	0.9999

Supplementary References

1. Yao, Z. *et al.* Extraordinary Separation of Acetylene-Containing Mixtures with Microporous Metal-Organic Frameworks with Open O Donor Sites and Tunable Robustness through Control of the Helical Chain Secondary Building Units. *Chem. Eur. J.* **16**, 5676-5683 (2016).
2. Chen, K.-J. *et al.* Benchmark C_2H_2/CO_2 and CO_2/C_2H_2 Separation by Two Closely Related Hybrid Ultramicroporous Materials. *Chem* **5**, 753-765 (2016).
3. Luo, F. *et al.* UTSA-74: A MOF-74 Isomer with Two Accessible Binding Sites per Metal Center for Highly Selective Gas Separation. *J. Am. Chem. Soc.* **17**, 5678-5684 (2016).
4. Li, Y.-P. *et al.* Ultramicroporous Building Units as a Path to Bi-microporous Metal-Organic Frameworks with High Acetylene Storage and Separation Performance. *Angew. Chem. Int. Ed.* **38**, 13590-13595 (2019).
5. Gao, J. *et al.* Mixed Metal-Organic Framework with Multiple Binding Sites for Efficient C_2H_2/CO_2 Separation. *Angew. Chem. Int. Ed.* **11**, 4396-4400 (2020).
6. Zhang, L. *et al.* Benchmark C_2H_2/CO_2 Separation in an Ultra-Microporous Metal-Organic Framework via Copper(I)-Alkynyl Chemistry. *Angew. Chem. Int. Ed.* **29**, 15995-16002 (2021).
7. Yang, L. *et al.* Adsorption Site Selective Occupation Strategy within a Metal-Organic Framework for Highly Efficient Sieving Acetylene from Carbon Dioxide. *Angew. Chem. Int. Ed.* **9**, 4570-4574 (2021).
8. Wang, L. *et al.* Interpenetration Symmetry Control Within Ultramicroporous Robust Boron Cluster Hybrid MOFs for Benchmark Purification of Acetylene from Carbon Dioxide. *Angew. Chem. Int. Ed.* **42**, 22865-22870 (2021).
9. Niu, Z. *et al.* A MOF-based Ultra-Strong Acetylene Nano-trap for Highly Efficient C_2H_2/CO_2 Separation. *Angew. Chem. Int. Ed.* **10**, 5283-5288 (2021).
10. Pei, J. *et al.* A Chemically Stable Hofmann-Type Metal-Organic Framework with Sandwich-Like Binding Sites for Benchmark Acetylene Capture. *Adv. Mater.* **24**, 1908275 (2020).
11. Peng, Y.-L. *et al.* Robust Ultramicroporous Metal-Organic Frameworks with Benchmark Affinity for Acetylene. *Angew. Chem. Int. Ed.* **34**, 10971-10975 (2018).
12. Li, P. *et al.* A Rod-Packing Microporous Hydrogen-Bonded Organic Framework for Highly Selective Separation of C_2H_2/CO_2 at Room Temperature. *Angew. Chem. Int. Ed.* **2**, 574-577 (2015).
13. Scott, H. S. *et al.* Highly Selective Separation of C_2H_2 from CO_2 by a New Dichromate-Based Hybrid Ultramicroporous Material. *ACS Appl. Mater. Interfaces* **39**, 33395-33400 (2017).
14. Lee, J. *et al.* Separation of Acetylene from Carbon Dioxide and Ethylene by a Water-Stable Microporous Metal-Organic Framework with Aligned Imidazolium Groups inside the Channels. *Angew. Chem. Int. Ed.* **26**, 7869-7873 (2018).
15. Ye, Y. *et al.* Pore Space Partition within a Metal-Organic Framework for Highly Efficient C_2H_2/CO_2 Separation. *J. Am. Chem. Soc.* **9**, 4130-4136 (2019).
16. Zeng, H. *et al.* Induced Fit of C_2H_2 in a Flexible MOF Through Cooperative Action of Open Metal Sites. *Angew. Chem. Int. Ed.* **25**, 8515-8519 (2019).

17. Xiang, S. *et al.* Open Metal Sites within Isostructural Metal-Organic Frameworks for Differential Recognition of Acetylene and Extraordinarily High Acetylene Storage Capacity at Room Temperature. *Angew. Chem. Int. Ed.* **27**, 4615-4618 (2010).
18. Qian, Q.-L. *et al.* A novel anion-pillared metal-organic framework for highly efficient separation of acetylene from ethylene and carbon dioxide. *J. Mater. Chem. A.* **14**, 9248-9255 (2021).
19. Zhang, Y. *et al.* A Microporous Metal-Organic Framework Supramolecularly Assembled from a Cu^{II} Dodecaborate Cluster Complex for Selective Gas Separation. *Angew. Chem. Int. Ed.* **24**, 8145-8150 (2019).
20. Zhang, Y. *et al.* Pillar iodination in functional boron cage hybrid supramolecular frameworks for high performance separation of light hydrocarbons. *J. Mater. Chem. A.* **48**, 27560-27566 (2019).
21. Zhang, Y. *et al.* Rational Design of Microporous MOFs with Anionic Boron Cluster Functionality and Cooperative Dihydrogen Binding Sites for Highly Selective Capture of Acetylene. *Angew. Chem. Int. Ed.* **40**, 17664-17669 (2020).
22. Chen, D.-M. *et al.* Tunable Robust pacs-MOFs: a Platform for Systematic Enhancement of the C_2H_2 Uptake and C_2H_2/C_2H_4 Separation Performance. *Inorg. Chem.* **5**, 2883-2889 (2018).
23. Burd, S. D. *et al.* Highly Selective Carbon Dioxide Uptake by [Cu(bpy-n)$_2$(SiF$_6$)] (bpy-1 = 4,4′-Bipyridine; bpy-2 = 1,2-Bis(4-pyridyl)ethene). *J. Am. Chem. Soc.* **8**, 3663-3666 (2012).
24. Cui, X. *et al.* Pore chemistry and size control in hybrid porous materials for acetylene capture from ethylene. *Science* **6295**, 141-144 (2016).
25. Jiang, M. *et al.* A thermostable anion-pillared metal-organic framework for C_2H_2/C_2H_4 and C_2H_2/CO_2 separations. *Chem. Eng. J.* 803-810 (2018).
26. Li, L. *et al.* Efficient separation of ethylene from acetylene/ethylene mixtures by a flexible-robust metal-organic framework. *J. Mater. Chem. A.* **36**, 18984-18988 (2017).
27. Wen, H.-M. *et al.* A microporous metal-organic framework with rare lvt topology for highly selective C_2H_2/C_2H_4 separation at room temperature. *Chem. Commun.* **26**, 5610-5613 (2015).
28. Hu, T.-L. *et al.* Microporous metal-organic framework with dual functionalities for highly efficient removal of acetylene from ethylene/acetylene mixtures. *Nat. Commun.* **1**, 7328 (2015).
29. Li, H. *et al.* Microporous Metal-Organic Framework with Dual Functionalities for Efficient Separation of Acetylene from Light Hydrocarbon Mixtures. *ACS Sustain. Chem. Eng.* **5**, 4897-4902 (2019).
30. Lin, R.-B. *et al.* Optimized Separation of Acetylene from Carbon Dioxide and Ethylene in a Microporous Material. *J. Am. Chem. Soc.* **23**, 8022-8028 (2017).
31. Chen, Y. *et al.* Boosting molecular recognition of acetylene in UiO-66 framework through pore environment functionalization. *Chem. Eng. Sci.* 116572 (2021).
32. Li, Q. *et al.* A Highly Connected Trinuclear Cluster Based Metal–Organic Framework for Efficient Separation of C_2H_2/C_2H_4 and C_2H_2/CO_2. *Inorg. Chem.* **18**, 13005-13008 (2020).
33. Qazvini, O. T. *et al.* Multipurpose Metal-Organic Framework for the Adsorption of

Acetylene: Ethylene Purification and Carbon Dioxide Removal. *Chem. Mater.* **13**, 4919-4926 (2019).
34. Xiang, S.-C. *et al.* Rationally tuned micropores within enantiopure metal-organic frameworks for highly selective separation of acetylene and ethylene. *Nat. Commun.* **1**, 204 (2011).
35. Yang, L. *et al.* A novel interpenetrated anion-pillared porous material with high water tolerance afforded efficient C_2H_2/C_2H_4 separation. *Chem. Commun.* **34**, 5001-5004 (2019).
36. Fan, W. *et al.* Tetrazole-Functionalized Zirconium Metal-Organic Cages for Efficient C_2H_2/C_2H_4 and C_2H_2/CO_2 Separations. *Angew. Chem. Int. Ed.* **32**, 17338-17343 (2021).
37. Sensharma, D. *et al.* The First Sulfate-Pillared Hybrid Ultramicroporous Material, SOFOUR-1-Zn, and Its Acetylene Capture Properties. *Angew. Chem. Int. Ed.* **8**, e202116145 (2021).
